# Metabolomics and Transcriptomics Reveal the Effects of Different Fermentation Times on Antioxidant Activities of *Ophiocordyceps sinensis*

**DOI:** 10.3390/jof11010051

**Published:** 2025-01-09

**Authors:** Min He, Tao Wang, Chuyu Tang, Mengjun Xiao, Xiaojian Pu, Jianzhao Qi, Yuling Li, Xiuzhang Li

**Affiliations:** 1 State Key Laboratory of Plateau Ecology and Agriculture, Qinghai Academy of Animal and Veterinary Science, Qinghai University, Xining 810016, China; himi1228@163.com (M.H.); 13085500761@163.com (T.W.); chuyutang0410@163.com (C.T.); 15574237597@163.com (M.X.); puxj@qhu.edu.cn (X.P.); yulingli2000@163.com (Y.L.); 2 Center of Edible Fungi, Northwest A&F University, Yangling, Xianyang 712100, China; qjz@nwafu.edu.cn

**Keywords:** untargeted metabolomics, *Ophiocordyceps sinensis*, LC-MS/MS, transcriptome, antioxidant activity

## Abstract

*Ophiocordyceps sinensis* is a fungus that is cultured through fermentation from wild Chinese cordyceps. While studies have examined its metabolites, the evaluation of its antioxidant capacity remains to be conducted. The antioxidant results of *O. sinensis* indicate that the ferric ion-reducing antioxidant power (FRAP), antioxidant capacity (2.74 ± 0.12 μmol Trolox/g), 2,2-diphenyl-1-picrylhydrazyl (DPPH•) free radical scavenging rate (60.21 ± 0.51%), and the hydroxyl free radical scavenging rate (91.83 ± 0.68%) reached a maximum on day 30. Using LC-MS/MS to measure the metabolites on D24, D30, and D36, we found that the majority of the differential accumulated metabolites (DAMs) primarily accumulate in lipids, organoheterocyclic compounds, and organic acids and their derivatives. Notably, the DAMs exhibiting high peaks include acetylcarnitine, glutathione, linoleic acid, and L-propionylcarnitine, among others. The transcriptome analysis results indicate that the differentially expressed genes (DEGs) exhibiting high expression peaks on D30 primarily included *lnaA*, *af470*, and *ZEB1*; high expression peaks on D24 comprised *SPBC29A3.09c* and *YBT1*; high expression peaks on D36 included *dtxS1*, *PA1538*, and *katG*. The combined analysis revealed significant and extremely significant positive and negative correlations between all the DAMs and DEGs. The primary enriched pathways (*p* < 0.05) included glutathione metabolism, tryptophan metabolism, carbon metabolism, biosynthesis of secondary metabolites, and phenylalanine metabolism. The metabolic pathway map revealed that the DAMs and DEGs influencing the antioxidant activity of *O. sinensis* were significantly up-regulated on D30 but down-regulated on D36. The correlation analysis suggests that an increase in the content of DEGs and DAMs promotes an increase in the levels of enzyme and non-enzyme substances, ultimately enhancing the antioxidant capacity of *O. sinensis*. These findings serve as a reference of how DAMs and DEGs affect the antioxidant activity of *O. sinensis*. This may contribute to the enhanced development and application of *O. sinensis*.

## 1. Introduction

Antioxidant is the abbreviation of antioxidant free radicals, which refers to substances that can effectively restrict the oxidation reaction of free radicals. Reactive oxygen species (ROS), the most prevalent oxygen free radicals in biological systems, can be generated naturally during normal cellular energy metabolism. Additionally, they may also form in response to various external stimuli and factors [1]. Research indicates that various chronic diseases in humans are associated with imbalances in ROS levels within the body [2]. When ROS are synthesized at appropriate levels, they can play a beneficial physiological role [3,4,5]. However, when ROS levels exceed a certain threshold, they can lead to oxidative stress [6,7], resulting in damage to numerous biological macromolecules, such as through lipid peroxidation [8,9,10]. This oxidative damage may further contribute to the development of diseases such as cancer, diabetes, and Alzheimer’s disease [11,12,13,14].

Antioxidants are a class of substances that can effectively prevent or delay auto-oxidation. They are defined as substances that can inhibit or delay the oxidation reaction of a substrate at a lower concentration than that of the oxidizable substrate, which may include saccharides, lipids, DNA, or proteins [15]. Antioxidants are currently categorized into two main types: natural and synthetic antioxidants. Natural antioxidants can further be subdivided into enzymatic antioxidants, which include glutathione, superoxide dismutase (SOD), catalase (CAT), and glutathione peroxidase (GPX), and non-enzymatic antioxidants, which comprise antioxidant enzyme cofactors, reactive oxygen species (ROS), and reactive nitrogen species (RNS) scavengers, as well as transition metal chelators [1,16]. Research indicates that the iron chelators pyoverdine and hydroxypyrid-4-ones possess significant free radical scavenging capabilities [17]. Various flavonoids, alkaloids, coumarins, and terpenoids derived from Chinese herbal medicine exhibit inhibitory effects on lipid peroxidation in mouse brain tissue [18,19,20]. Soybean isochalcones demonstrate a pronounced ability to enhance antioxidant enzyme activity in mice, thereby preventing the infiltration of free radicals and inhibiting lipid peroxidation [21]. Glutathione is crucial in managing free radicals, serving as an antioxidant and nutrient, and plays a vital role in maintaining metabolic balance [22]. It has been reported that natural antioxidants are generally non-toxic and rarely cause harmful side effects. Consequently, the search for antioxidants—particularly phenolic derivatives, peptides/protein hydrolysates, phospholipids, and polysaccharides—from natural sources such as plants, bacteria, and fungi has emerged as a significant focus for enhancing the body’s antioxidant capacity. This effort is crucial in alleviating oxidative stress and preventing various diseases. Furthermore, the implementation of effective strategies in both the food industry and preventive medicine is showing a notable upward trend [23,24].

Chinese cordyceps, recognized as a unique “food and medicine homologous” cordyceps [25], exhibit a range of pharmacological effects, including antioxidant [26], immune regulation [27], and anti-cancer properties [28]. Consequently, the consumption of such cordyceps can help humans to reduce problems such as oxidative stress, all while alleviating concerns regarding potential side effects and health risks associated with synthetic antioxidants. However, wild Chinese cordyceps primarily inhabit the elevations exceeding 3000 m above sea level [29], and the extreme environmental conditions make its resources scarce [30]. High market demand [31,32] and the challenges associated with artificial cultivation [33] have prompted researchers to employ modern biological fermentation technology to cultivate *O. sinensis* [34]. As an asexual fungus of Chinese cordyceps [35,36], *O. sinensis* shares similar active ingredients [37] and pharmacological effects [38] with its counterpart. Research indicates that *O. sinensis* has beneficial effects on blood pressure, blood sugar levels, inflammation, and oxidative stress [39]. It has been shown to enhance the antioxidant capacity in immunosuppressed mice [40]. *O. sinensis* polysaccharides exhibit specific immunological activities [41]. Furthermore, *O. sinensis* melanins can decrease intracellular ROS levels and mitigate oxidative damage to cells induced by peroxide (H_2_O_2_) [42]. Nutraceuticals, specifically ‘Bailing’ capsules made from the fermented powder of *O. sinensis*, have gained significant popularity in the market [43]. These studies not only highlight the potential connections and similarities among these fungi but also suggest that *O. sinensis* may serve as a superior alternative to wild Chinese cordyceps.

Research has demonstrated that the metabolite levels of Chinese cordyceps fluctuate based on growth conditions, environmental factors, and storage duration. These variations may lead to differences in pharmacological effects [44,45]. Consequently, it is essential to investigate the fermented mycelium of Chinese cordyceps. Currently, studies have reported variations in the metabolites of *O. sinensis* [46,47]. However, the impact of different fermentation durations on the metabolites and antioxidant capacity of *O. sinensis* has been seldom investigated. The present study utilized 2,2-diphenyl-1-picrylhydrazyl radical scavenging ability (DPPH), ferric ion-reducing antioxidant power (FRAP), hydroxyl free radical scavenging capacity (•OH), and superoxide anion radical scavenging capacity (O_2_^•−^) to characterize the antioxidant activity of *O. sinensis* at different fermentation times. The antioxidant activities of *O. sinensis* were assessed to elucidate the impact of varying fermentation durations on its antioxidant capacity. By employing non-targeted metabolomics and transcriptomics to investigate the metabolites and gene expression of *O. sinensis* across different fermentation times, we aimed to determine whether the observed differences in antioxidant activity were associated with differential metabolites and genes. This study seeks to elucidate the roles of these differential metabolites and genes in antioxidant activity, thereby providing a theoretical basis for the development of novel and efficient antioxidants.

## 2. Materials and Methods

### 2.1. Materials

The *O. sinensis* strain was isolated from wild Chinese cordyceps collected in Guide County, Hainan Prefecture, Qinghai Province (36°21′34″ N 101°25′46″ E, altitude: 3797 m). It was stored in the Chinese cordyceps Laboratory of the Qinghai Academy of Animal and Veterinary Science at Qinghai University. Following activation, the strain was inoculated into an Erlenmeyer flask containing 100 mL of liquid (50% beef broth, 0.5% peptone, 0.15% yeast extract, 3% glucose, 0.02% MgSO_4_, and 0.2% KH_2_PO_4_), and it was fermented and cultured on a rotating shaker at 18 °C and 135 rpm. Mycelia was harvested at 18, 21, 24, 27, 30, 33, 36, and 39 days, respectively. The samples were centrifuged at 4 °C at 10,000× *g* for 20 min, washed 3–4 times with deionized water, and the clean mycelia were collected. The fresh weight was measured, and the samples were stored at −80 °C for further analysis.

### 2.2. Determination of Antioxidant Activity

The kits for ferric ion-reducing antioxidant power (FRAP), 2,2-diphenyl-1-picrylhydrazyl radical scavenging ability (DPPH•), hydroxyl free radical scavenging capacity (•OH), superoxide dismutase (SOD), peroxidase (POD), catalase (CAT), glutathione peroxidase (GSH-Px), flavonoids, and polysaccharides were sourced from Suzhou Keming Biotechnology Co., Ltd., Suzhou, China, whereas a superoxide anion radical scavenging capacity (O_2_^•−^) kit was acquired from Beijing Solarbio Technology Co., Ltd., Beijing, China. Each assay index was conducted meticulously, following the provided instructions.

### 2.3. Untargeted Metabolomics Analysis

#### 2.3.1. QC Sample Preparation and Metabolites Extraction

We took 0.1 g of tissue from each of the two samples and added it to 1 mL of mixed solution (chromatographically pure, LC-MS-grade methanol, Fisher, https://corporate.thermofisher.com; chromatographically pure, LC-MS-grade, acetonitrile, Merck, https://www.merck.com—aqueous = 2:2:1 (*v*/*v*)), respectively). The solution was vortexed for 30 s to ensure its homogeneity. The mixture was sonicated in ice water (KQ-250DE Ultrasonic Instrument, Kunshan Ultrasonic Instrument Co., Ltd., Kunshan, China) for 30 min and then left to stand at −20 °C for 10 min. The solution was centrifuged (low-temperature, high-speed centrifuge, 5430R Eppendorf) at 14,000× *g* and 4 °C for 20 min, and then 500 μL of the supernatant from this solution was vacuum dried. Six replicates were performed for each sample. In the mass spectrometry analysis, the 18 dried samples were redissolved by adding them to 100 μL of acetonitrile solution (acetonitrile/water = 1:1, *v*/*v*). Then, they were swirled for 30 s, sonicated in ice water for 30 min, and centrifuged at 14,000× *g* and 4 °C for 15 min. Finally, 120 μL of the supernatant was used for the LC-MS/MS analysis. We took 10 μL of liquid from each of the two samples and combined them to create a quality control (QC) sample. The QC sample was inserted to assess the status of the instrument and to ensure data reliability before formal testing.

#### 2.3.2. LC-MS/MS and Mass Spectrum Condition

The samples were separated by an Agilent 1290 Infinity LC ultra-high performance liquid chromatography (Agilent, Santa Clara, CA, USA) HILIC column. The column temperature was 25 °C; flow rate was 0.5 mL/min; sample size was 2 μL; mobile phase composition A: water +25 mM ammonium acetate (NH4AC, purity ≥ 99%, Sigma Aldrich, Saint Louis, MI, USA, https://www.sigmaaldrich.cn) +25 mM ammonia water (NH4OH, purity ≥ 99%, Fisher, Waltham, MA, USA), B: acetonitrile; samples were placed in a 4 °C automatic injector throughout the entire analysis process. The samples were analyzed continuously in random order. The gradient separation procedure is shown in Table 1.

The positive and negative ESI source conditions of the AB Triple TOF 6600 mass spectrometer (Shanghai Applied Protein Technology Co., Ltd., Shanghai, China) after HILIC chromatographic separation were as follows: Ion Source Gas1 (Gas1): 60, Ion Source Gas2 (Gas2): 60, curtain gas (CUR): 30; source temperature: 600 °C; IonSapary Voltage Floating (ISVF) ± 5500 V; TOF MS scan *m*/*z* range: 60–1000 Da; product ion scan *m*/*z* range: 25–1000 Da; TOF MS scan accumulation time 0.20 s/spectra; product ion scan accumulation time 0.05 s/spectra; sheath gas flow rate, 35 arbitrary units; auxiliary gas flow rate, 8 arbitrary units; secondary mass spectrometry was performed using information-dependent acquisition (IDA) and high-sensitivity mode; declustering potential (DP): ±60 V (positive and negative modes); collision energy: 35 ± 15 eV. The IDA settings were as follows: exclude isotopes within 4 Da; candidate ions to monitor per cycle: 10.

#### 2.3.3. Data Processing and Statistical Analyses

The peak alignment, retention time correction, and peak area extraction of the raw data were ascertained using XCMS software (XCMS online 3.7.1). From the data obtained from XCMS extraction, metabolites with missing values of more than 50% within the group were removed and were not involved in subsequent analyses. The missing data were filled in by using the KNN (K-Nearest Neighbor) method, and extreme values were removed. Finally, the data were normalized for total peak area to ensure that parallelism could be compared between samples and metabolites.

The raw data were processed based on information from the Kyoto Encyclopedia of Genes and Genomes database (KEGG, https://www.genome.jp/kegg/pathway.html, accessed on 15 March 2024) and the Human Metabolome Database (HMDB, http://www.hmdb.ca, accessed on 15 March 2024) for compound annotation. Unsupervised and supervised dimensionality reduction methods were utilized to conduct principal component analysis (PCA) and orthogonal partial least squares discriminant analysis (OPLS-DA) using the R package model (http://www.r-project.org/). Differential metabolites were identified based on the variable weight value (VIP) and *p* value from the (O)PLS model, with metabolites of VIP > 1 and *p* < 0.05 considered to be differential.

### 2.4. Transcriptome Analysis

#### 2.4.1. RNA Extraction

We took 0.1 g of mycelium and a small amount of liquid nitrogen (Guangzhou Zhenyu Weiou Trading Co., Ltd., Guangzhou, China) and ground them thoroughly in a mortar, and then transferred the mixture to a 1.5 mL centrifuge tube. To the mixture, 1 mL of Trizol reagent (Life Technologies, Carlsbad, CA, USA) was added and mixed thoroughly before being left to stand at room temperature for 10 min. Subsequently, 200 µL of chloroform (Guangzhou Chemical Reagent Factory, Guangzhou, China) was added to the mixture and mixed thoroughly. The solution was centrifuged (Eppendorf 5427R, Eppendorf AG, Hamburg, Germany) at 12,000 rpm and 4 °C for 10 min. We carefully collected the upper aqueous phase and added an equal volume of phenol (Guangzhou Chemical Reagent Factory)/chloroform (25:24). The solution was mixed thoroughly before centrifuging at 4 °C and 12,000 rpm for 10 min. After taking the upper aqueous phase of the mixture and adding an equal volume of isopropanol alcohol (Guangzhou Chemical Reagent Factory), the solution was allowed to stand at −20 °C for 1 h. Then, the solution was centrifuged at 4 °C and 12,000 rpm for 10 min. After the supernatant of the solution was sucked out and discarded, 1 mL of 75% ethanol (Guangzhou Chemical Reagent Factory) was added and precipitated for 5 min, and the mixture was centrifuged at 4 °C and 8000 rpm for 5 min. The ethanol in the solution was pipetted (Eppendorf Research Plus, Eppendorf AG, Germany) and the centrifuge tubes were dried in a vacuum dryer (Eppendorf concentrator Plus, Eppendorf AG, Germany) for 2–4 min. A volume of 20–50 μL RNase-free water was added to a dried centrifuge tube and the mixture was dissolved at room temperature for 10 min; then, the liquid was mixed and centrifuged for 2 min. Finally, 1 mL of liquid was taken to measure the concentration, mass, and integrity of the RNA using a microspectrophotometer (Thermo Fisher Scientific, Waltham, MA, USA, Nanodrop 2000).

#### 2.4.2. RNA Library Construction

After the RNA extraction was completed, the mRNA Capture Beads were removed from the refrigerator and allowed to stand at room temperature for approximately 30 min. The nuclease-free centrifuge tube containing 10 ng to 4 μg of total RNA was rehydrated to 50 μL and set aside on ice. All library construction steps were conducted strictly according to the provided instructions. After, First Strand cDNA synthesis was carried out, and we took 6 μL Strand Specificity Reagent, 2 μL 1st Strand Enzyme Mix, 17 μL Fragmented mRNA, and 25 μL of the total sample volume and mixed them. The mixture was placed in a preheated PCR instrument for reaction (cover temperature: 105 °C) under the following conditions: 25 °C, 10 min; 42 °C, 15 min; 70 °C, 15 min; 4 °C, hold. Then, for Second Strand cDNA synthesis, we took 25 μL 1st Strand cDNA, 30 μL 2nd Strand Buffer (dNTP or dUIP), 5 μL 2nd Strand Enzyme Master Mix, and 60 μL of the total sample volume and mixed them. The mixture was placed in a preheated PCR instrument (cover temperature: 105 °C) under the following conditions: 16 °C, 30 min; 72 °C, 15 min; 4 °C, hold. Ligation product purification, PCR amplification, and amplification product bead purification were performed using Hieff NGS^®^ DNA Selection Beads (Yeasen Biotechnology (Shanghai) Co., Ltd., Shanghai, China). Finally, the High-Sensitivity DNA Assay Kit (Agilent Technologies, Agilent Technologies Ltd., Santa Clara, CA, USA, 5067-4626) was selected to quantify and check the quality of the samples for library quantification and complete the RNA library construction.

#### 2.4.3. Quantification, Differential Expression Analysis, and Enrichment Analysis of Genes

The raw gene data in FASTQ format contain a substantial number of low-quality reads. We utilized the fast (0.22.0) software to filter the raw data, thereby obtaining high-quality clean reads for subsequent analysis. RSEM (v2.15) was employed to calculate and compare the expression levels of all genes in each sample, with FPKM utilized for normalization of these expression levels. Utilizing the gene expression data, R (http://www.r-project.org/) facilitated the execution of principal component analysis (PCA). DESeq2 (v1.38.3) was applied to identify differentially expressed genes between groups, with filtering criteria set to an expression difference fold of |log2FoldChange| > 1 and a *p* value < 0.05. All genes were mapped to the GO database (Gene Ontology, http://www.geneontology.org) and GO (v2.50.0) was used to conduct GO enrichment analysis on the differentially expressed genes, calculating the number of differentially enriched genes for each term. The hypergeometric test was employed to determine the *p* value (*p* < 0.05) and to identify GO terms that are significantly enriched among the differentially expressed genes, thereby elucidating the primary biological functions associated with these genes. The Cluster Profiler (v4.6.0) software was utilized to conduct KEGG pathway enrichment analysis (Kyoto Encyclopedia of Genes and Genomes, https://www.genome.jp/kegg/pathway.html, accessed on 30 October 2024) on differentially expressed genes (*p* < 0.05) to identify significant enrichment pathways. Additionally, the R package was employed to calculate the correlation between DAMs and DEGs. Pearson’s correlation coefficient came closer to 1, indicating a stronger correlation between DAMs and DEGs.

#### 2.4.4. Quantitative Real-Time Polymerase Chain Reaction (qRT-PCR) Verification

To validate the RNA-seq data, we selected 11 genes for qRT-PCR validation. The ACT gene was utilized as a standardized expression gene, verified by qRT-PCR [37]. SPSS 26.0 software was employed to analyze the correlation and significance of the data related to antioxidant activity, while Origin 2024 was used to create a heat map illustrating the correlation of antioxidant activity.

## 3. Results

### 3.1. Antioxidant Activity

**Figure 1:**
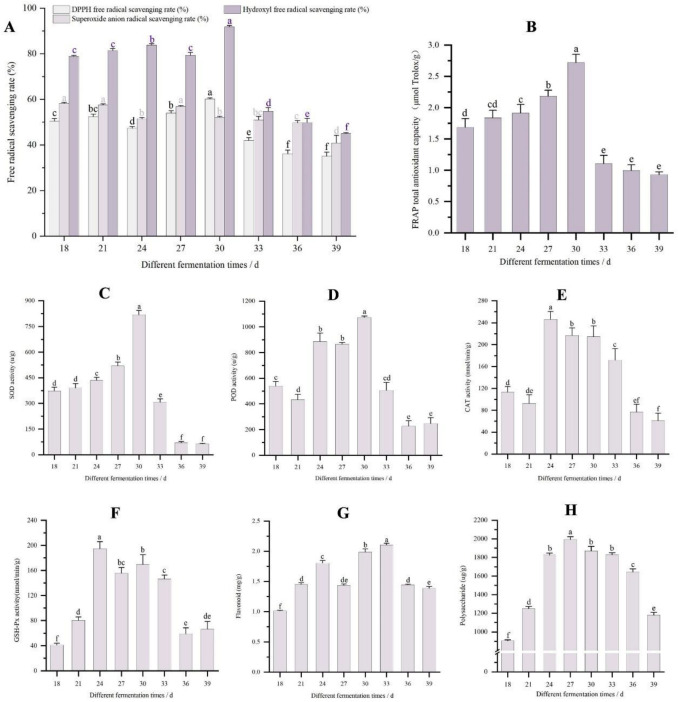
*Ophiocordyceps sinensis* at different fermentation times. (**A**) Antioxidant activity of 2,2-diphenyl-1-picrylhydrazyl radical scavenging rate (DPPH•), hydroxyl free radical scavenging rate (•OH), and superoxide anion radical scavenging rate (O_2_^•−^). (**B**) Antioxidant activity of ferric ion-reducing antioxidant power (FRAP). (**C**) Changes in SOD activity with *O. sinensis* at different fermentation times. (**D**) Changes in POD activity with *O. sinensis*. (**E**) Changes in CAT activity with *O. sinensis*. (**F**) Changes in GSH-Px activity with *O. sinensis*. (**G**) Changes in flavonoid with *O. sinensis*. (**H**) Changes in polysaccharide with *O. sinensis*. Analysis of Variance (ANOVA) shows that different lowercase letters indicate significant differences between samples at the 0.05 level.

In this study, we used the method of FRAP, DPPH•, •OH, and O_2_^•−^ to determine the antioxidant activity of *O. sinensis* on D24, D30, and D36. The results indicate that the FRAP (2.74 ± 0.12 μmol Trolox/g), DPPH• (60.21 ± 0.51%), and •OH (91.83 ± 0.68%) all reached their maximum values at 30 days. The O_2_^•−^ on D18, D21, and D27 were significantly higher than at the other fermentation times, and the difference between these three was not significant (Figure 1A,B). The activities of SOD, POD, CAT, and GSH-Px increased significantly on D24, but these parameters showed a significant decrease by D33. The flavonoid and polysaccharide contents were higher on D33 and started to decrease on D36 (Figure 1C–H). We utilized FRAP as the primary indicator and comprehensively evaluated the other three antioxidant indicators. Our findings reveal that the antioxidant activity throughout the fermentation cycle of *O. sinensis* initially increased and then decreased. Notably, the antioxidant activity of *O. sinensis* exhibited a significant increase on D24, reaching its peak on D30 before beginning to decline on D33. Furthermore, the antioxidant activity between D36 and D39 was not significantly different. Consequently, we selected three samples (D24, D30, and D36) with significant differences (*p* < 0.05) for subsequent analysis.

### 3.2. Metabolomic Analysis

#### 3.2.1. Metabolite Profiles Analysis

This study employed LC-MS/MS technology for non-targeted metabolomics to obtain the total ion chromatograms (TIC) of QC, D24, D30, and D36. The results of the QC retention time and peak intensity overlap indicate that the mass spectrometer maintains a stable signal when detecting the same sample at different time points (see Supplementary Figure S1A for details). Notably, significant differences were observed in the peak heights and positions on D24, D30, and D36 during the two time intervals of 0.8–3.4 min and 3.8–8.6 min (See Supplementary Figure S1B for detailed information).

**Figure 2:**
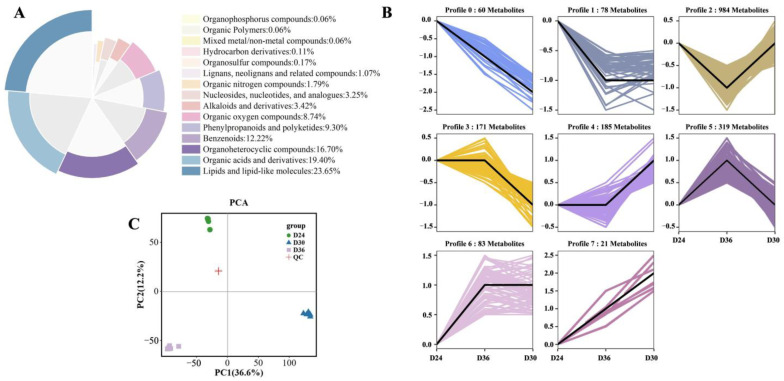
The metabolites detected on D24, D30, and D36 samples. (**A**) Metabolite superclass classification on D24, D30, and D36. (**B**) Cluster analysis of all metabolites is presented, with the abscissa representing various fermentation times and the ordinate indicating the relative content of metabolites. (**C**) PCA of D24, D30, D36, and QC.

To elucidate the metabolic profile of *O. sinensis* at varying fermentation durations, we employed LC-MS/MS technology in conjunction with public databases such as Mass Bank, Metlin, and MoNA, alongside a custom-built secondary mass spectrometry database. This approach facilitated the detection of metabolites from samples on D24, D30, and D36. The findings revealed a total of 1903 metabolites, comprising 1171 ESI+ metabolites and 732 ESI- metabolites. These metabolites were categorized into 15 supercategories, predominantly including lipids and lipid-like molecules, organic acids and derivatives, organoheterocyclic compounds, benzenoids, phenylpropanoids, polyketides, and organic oxygen compounds, among others. Notably, lipids and lipid-like molecules constituted the largest group, accounting for 23.65% of the total, followed by organic acids and derivatives (19.40%), organoheterocyclic compounds (16.70%), and benzenoids (12.22%) (Figure 2A). Additionally, the Short Time-series Expression Miner software (https://www.omicshare.com/tools/home/report/reporttrend.html, accessed on 31 October 2024) was employed to analyze the variations in the content of 1903 metabolites on D24, D30, and D36. Based on their accumulation patterns, we identified eight metabolite profiles. Notably, significant differences were observed in the changes in metabolite content among these profiles across the three fermentation times. Specifically, the alterations in the metabolite content of profiles 2, 4, and 7 exhibit a trend analogous to the changes in antioxidant capacity as measured by FRAP, initially increasing before subsequently decreasing (Figure 2B).

#### 3.2.2. Multivariate Statistical Analysis

The principal component analysis (PCA) highlights the differences among D24, D30, D36, and QC, as well as the variability among samples within each group. The PCA plot indicates that the contribution rate of PC1 is 36.6%, while that of PC2 is 12.2%. This suggests that the intra-group repeatability of each sample is satisfactory. Notably, the samples within each group exhibit a clear trend of separation, indicating significant differences in the metabolites on D24, D30, and D36. QC is clustered near the center of the PCA plot, which suggests that the measurement method is stable and that the metabolomic data for each sample are reliable (Figure 2C). The OPLS-DA results demonstrate that all sample groups within each model fall within the confidence zone, further supporting the reliability of the model (Figure S2A). The permutation test random model reveals that the Q^2^ intercepts generated for the three groups of samples are 0.05, −0.02, and −0.00, respectively (all < 0.5). This indicates that the original model is not overfitted and exhibits good stability (Figure S2B).

#### 3.2.3. Screening of Differential Accumulated Metabolites (DAMs)

The VIP of the first principal component of the OPLS-DA model quantifies the extent to which each metabolite influences the sample. The *p* < 0.05 threshold and VIP > 1 criterion from the *t*-test were employed to identify differential metabolites and auxiliary marker metabolites across the groups. The results of the volcano chart visualization indicate that there are 867 DAMs between D24 and D30 (501 up-regulated and 366 down-regulated), 970 DAMs between D24 and D36 (312 up-regulated and 658 down-regulated), and 1015 DAMs between D36 and D30 (670 up-regulated and 345 down-regulated) (Figure S8). In the comparison of D24 versus D30, we observed that lipid compounds, organic acids and their derivatives, organic oxygen compounds, nucleosides and their analogs, and organic nitrogen compounds were significantly enriched on D24. Conversely, organoheterocyclic compounds, benzenoids, alkaloids and their derivatives, and phenylpropanoids and polyketides were significantly enriched on D30. In the comparison of D24 versus D36, we found that all superclass compounds were significantly enriched on D24. Lastly, in the comparison of D36 versus D30, we noted that only organic nitrogen compounds and organosulfur compounds were significantly enriched on D36, while the levels of other compounds were higher on D30 compared to D36 (Figure S3).

**Figure 3:**
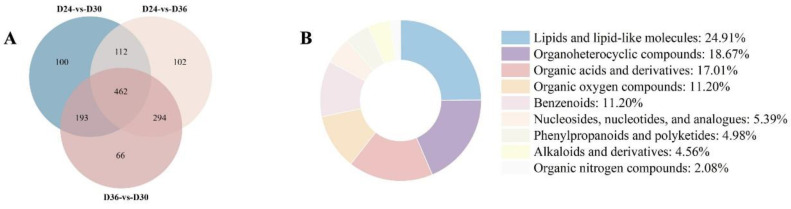
The differential accumulated metabolites (DAMs) analysis on D24, D30, and D36. (**A**) Venn diagram of metabolites detected on D24 vs. D30, D24 vs. D36, and D36 vs. D30. (**B**) Superclass classification of the 462 key metabolites.

Statistical analysis was performed of shared and unique metabolites between three comparison groups using Venn diagrams. The results illustrate that there are 462 overlapping DAMs among D24, D30, and D36 (Figure 3A), which can be categorized into nine superclasses, including lipids such as linoleic acid, behenic acid, and dodecanoic acid; and amino acids such as L-alanine, L-homoarginine, and lysine, among others. Among these nine supercategories, the top three are lipids, organoheterocyclic compounds, and organic acids and derivatives (Figure 3B). Consequently, DAMs within these three superclasses may serve as potential biomarkers for distinguishing differences in the antioxidant activity of *O. sinensis* at various fermentation times (D24, D30, and D36).

#### 3.2.4. Identification of Key Differential Accumulated Metabolites (DAMs)

**Figure 4:**
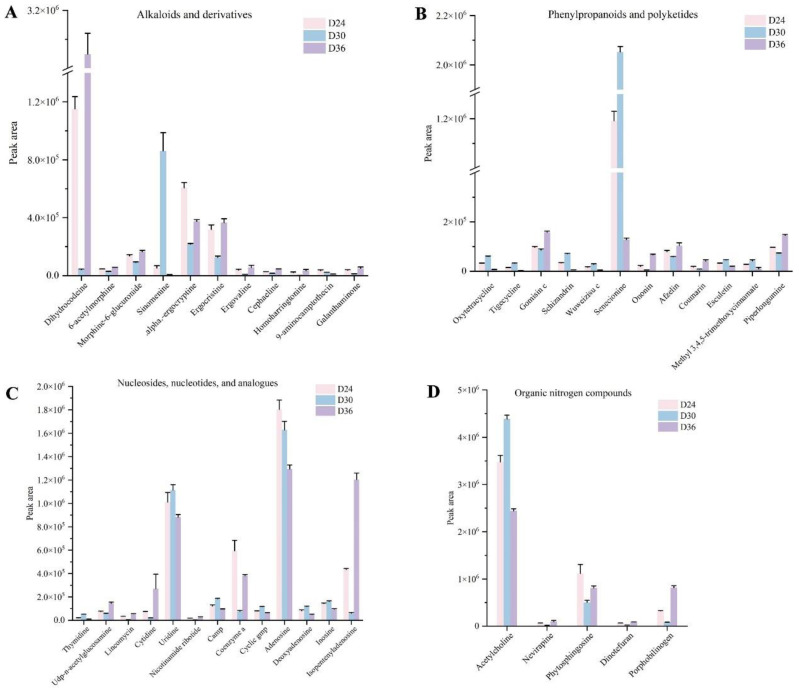
Changes in the peak area of differential accumulated metabolites (DAMs) for different superclasses. The DAMs of (**A**) alkaloids and derivatives, (**B**) phenylpropanoids and polyketides, (**C**) nucleosides, nucleotides, and analogues, (**D**) organic nitrogen compounds detected in *O. sinensis* at different fermentation times (D24, D30, and D36). The horizontal coordinate represents the DAMs, and the vertical coordinate is the peak area of the DAMs.

By examining the peak areas of the differential metabolites across the nine superclasses at fermentation times D24, D30, and D36, we identified DAMs that exhibit high expression levels and significant differences. The results indicate that there are nine DAMs that significantly differ within the superclass of lipids and lipid-like molecules. These include linoleic acid, linolenic acid, L-propionylcarnitine, isobutyryl-L-carnitine, erucamide, acetylcarnitine, glycerophosphocholine, 2-oleoyl-1-palmitoyl-sn-glycero-3-phosphocholine, and 1-stearoyl-2-oleoyl-sn-glycero-3-phosphoethanolamine. Notably, peaks on D30 show that acetylcarnitine is the only DAM with the highest abundance (Figure S4A). Significant differences were observed in six DAMs within the organic acids and derivatives superclass, specifically including guanidinoethyl sulfonate, taurine, scopolamine, benalaxyl, betaine, and prilocaine. Among these, the peak values of guanidinoethyl sulfonate, taurine, benalaxyl, L-pyroglutamic acid, glutathione, and gly-leu on D30 were significantly higher than those on D24 and D36 (Figure S4B). In the organoheterocyclic compounds, there was a greater abundance of DAMs exhibiting significant differences. Notably, DAMs with peak values exceeding those on D24 and D36 include meperidine, ezetimibe, N-acetyl-5-hydroxytryptamine, N-methyltryptamine, and olaparib. Conversely, DAMs with peak values greater than those on D30 primarily include D-neopterin, triptolide, paxilline, dimethenamid, amobarbital, nefazodone, and p-aminoazobenzene (Figure S4C). There were eight high-content and significantly different DAMs in benzenoids and five in organic oxygen compounds. These include dobutamine, naltrexone, 3,3′-dimethoxybenzidine, amprenavir, cyproheptadine, pyridaben, terfenadine, luminespib, d-psicose, simmondsin, d-threitol, gentiopicroside, and trehalose. With the exceptions of d-psicose and d-threitol, the peak values of the other DAMs on D24 and D36 were higher than those observed on D30 (Figure S4D,E). Among the remaining superclasses, we identified that DAMs with peak values higher than on D24 and D36 included sinomenine, senecionine, uridine, and acetylcholine. Additionally, there were eight DAMs with peak values exceeding D30, which include dihydrocodeine, alpha-ergocryptine, ergocristine, coenzyme a, adenosine, isopentenyladenosine, phytosphingosine, and porphobilinogen (Figure 4).

#### 3.2.5. KEGG Pathway Analysis

The KEGG pathway database was utilized to enrich the metabolic pathways of DAMs on D24, D30, and D36, with the top 20 pathways (*p* < 0.05) selected for analysis. The results indicated that monobactam biosynthesis, sphingolipid, and tryptophan were the top three metabolism pathways that exhibited significant differences on D24 vs. D30. In contrast, the top three pathways with significant differences between D24 and D36 were microbial metabolism in diverse environments, diterpenoid biosynthesis, and starch and sucrose metabolism. The top three metabolism pathways that exhibited significant differences on D36 vs. D30 were sphingolipid, sulfur, and carbapenem biosynthesis (Figure S5).

We identified that lysine degradation, carotenoid biosynthesis, and the biosynthesis of alkaloids derived from ornithine, lysine, and nicotinic acid are metabolic pathways shared among the three samples. The results of metabolic pathway enrichment indicated that the biosynthesis of alkaloids derived from ornithine, lysine, and nicotinic acid displayed significant differences among the three comparison groups: D24 vs. D30, D24 vs. D36, and D36 vs. D30 (*p* < 0.05).

### 3.3. Transcriptomic Analysis

#### 3.3.1. Transcriptome Sequencing Analysis

To investigate the impact of varying fermentation times (D24, D30, D36) on the gene expression of *O. sinensis*, this study performed three repeated transcriptome sequencing analyses on three samples. Following the removal of low-quality reads, a total of 372,304,594 clean reads and 54,869,547,953 base pairs of clean data were obtained. The GC content varied between 59.12% and 60.17%, while the lowest percentages for Q20 and Q30 were recorded at 98.06% and 94.73%, respectively (refer to Supplementary Table S1). These results indicate that the sequencing data for the three samples are of high quality and are suitable for further analysis.

**Figure 5:**
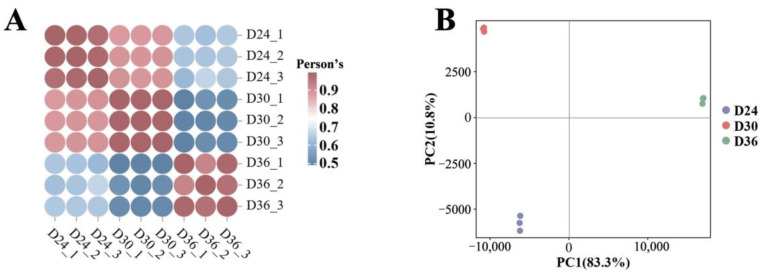
Principal component analysis and correlation analysis of 3 samples. (**A**) Sample correlation test. Redder colors indicate higher correlation; bluer colors indicate lower correlation. (**B**) PCA.

To ensure the reliability of transcriptome sequencing data, we performed a correlation evaluation of gene expression levels among biological replicates. The results indicated that the biological replicates of each sample exhibited a high Pearson’s correlation coefficient, suggesting good reproducibility. Notably, the correlation coefficient between D24 and D30 was high (Pearson’s > 0.8), while the correlation coefficients for the comparison groups D24 vs. D36 and D36 vs. D30 were lower (Pearson’s < 0.8) (Figure 5A). Additionally, the PCA plot revealed that the contribution rate of PC1 was 83.3%, and that of PC2 was 10.8%. This indicates strong aggregation and repeatability within each sample group. Furthermore, the samples in each group displayed a clear trend of separation, highlighting significant differences among D24, D30, and D36 (Figure 5B). Overall, both the correlation and PCA demonstrate that the transcript sequencing data from the three samples are reliable and suitable for further analysis.

#### 3.3.2. Differentially Expressed Genes (DEGs) of Three Samples

**Figure 6:**
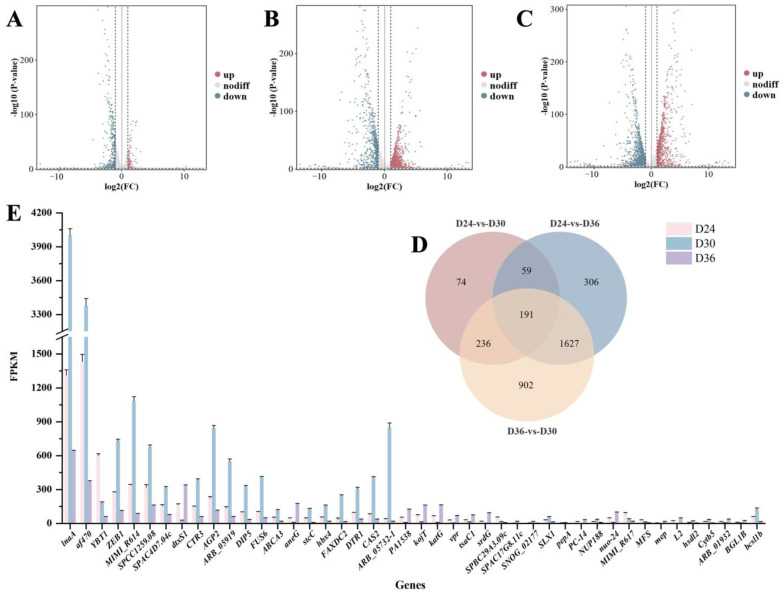
Identification of differentially expressed genes. (**A**) D24 vs. D30, (**B**) D24 vs. D36, (**C**) D36 vs. D30, (**D**) Venn diagrams for 3 comparison groups. (**E**) The differentially expressed genes (DEGs) detected in *O. sinensis* at different fermentation times (D24, D30, and D36). The horizontal coordinate represents the DEGs, and the vertical coordinate is the peak area of the DEGs.

The differential gene expression profiles of the three samples were compared based on the thresholds of |log2 Fold Change| > 2 and *p* value < 0.05. The results indicate that 560 DEGs were identified in the comparison of D24 vs. D30, comprising 114 up-regulated and 446 down-regulated genes (Figure 6A). In the comparison of D24 vs. D36, 2183 DEGs were found, including 1476 up-regulated and 707 down-regulated genes (Figure 6B). The comparison of D36 vs. D30 revealed 2956 DEGs, with 1003 up-regulated and 1953 down-regulated genes (Figure 6C). The Venn diagram analysis demonstrated that the three comparison groups contained 74, 306, and 902 unique DEGs, respectively, resulting in a total of 191 DEGs. Following the screening of these 191 DEGs, we identified 44 effective DEGs (Figure 6D). The Fragments Per Kilobase of exon model per Million mapped fragments (FPKM) values of 44 DEGs on D24, D30, and D36 were analyzed, leading to the identification of DEGs with high expression levels and significant differences. The results indicated that 17 DEGs exhibited higher expression levels on D30 compared to on D24 and D36. These genes primarily included *lnaA*, *af470*, *ZEB1*, *MIMI_R614*, *SPCC1259.08*, *SPAC4D7.04c*, *CTR3*, *AGP2*, *DIP5*, *ABCA3*, *stcC*, *hbx4*, *FAXDC2*, *DTR1*, *CAS2*, *ARB_05732-1*, and *bcsl1b*. Additionally, the genes exhibiting higher expression peaks on D24 included *SPBC29A3.09c* and *YBT1*, while those with higher peaks on D36 comprised *dtxS1*, *PA1538*, *kojT*, *katG*, *vpr*, *tsaC1*, *srdG*, *nuo-24*, and *aneG* (Figure 6E); see Table S3 for gene details.

#### 3.3.3. GO Enrichment Analysis and KEGG Enrichment Analysis

The DEGs from the three comparison groups were classified through GO enrichment analysis and KEGG enrichment analysis to elucidate their biological functions. The results of the GO enrichment analysis indicated that all the DEGs were mainly labeled as three main categories: molecular function, biological process, and cellular component. Specifically, 1606, 7068, and 11,109 DEGs were enriched in the comparisons of D24 vs. D30, D24 vs. D36, and D36 vs. D30, respectively. An analysis of the up- and down-regulation relationships revealed that a limited number of the DEGs were up-regulated in the D24 vs. D30 comparison, with their associated GO terms primarily encompassing molecular transducer activity and biological adhesion (Figure S6A). On D24 vs. D36, nine GO terms were significantly up-regulated, including cellular processes, metabolic processes, biological regulation, localization, cellular anatomical entities, catalytic activity, binding, transporter activity, and transcription regulator activity (Figure S6B). On D36 vs. D30, nine GO terms were significantly up-regulated, including regulation of biological processes, response to stimulus, developmental processes, negative regulation of biological processes, multicellular organismal processes, positive regulation of biological processes, immune system processes, protein-containing complex activity, and structural molecule activity (Figure S6C).

Based on the KEGG database, the DEGs were mapped, and the top 20 KEGG pathways (*p* < 0.05) were selected for analysis to investigate the impact of varying culture times on the enrichment of *O. sinensis* DEGs pathways. The results indicate that the DEGs in the comparison of D24 vs. D30 were predominantly significantly enriched in KEGG pathways related to pentose and glucuronate interconversions, basal transcription factors, and galactose metabolism (Figure S7A). In the D24 vs. D36 comparison, the DEGs were primarily significantly enriched in pathways associated with glycine, serine, and threonine metabolism; 2-oxocarboxylic acid metabolism; and pantothenate and CoA biosynthesis (Figure S7B). Furthermore, the DEGs in the D36 vs. D30 comparison were mainly significantly enriched in KEGG pathways related to phenylalanine metabolism, pantothenate and CoA biosynthesis, and tyrosine metabolism (Figure S7C).

### 3.4. Joint Analysis of the Transcriptome and Metabolome

#### 3.4.1. Correlations Analysis

**Figure 7:**
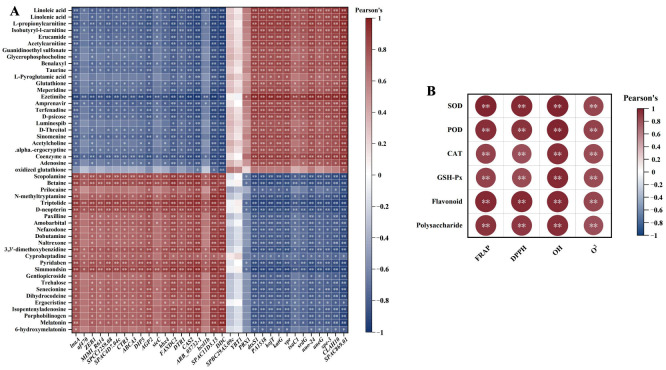
Correlation clustering heatmap. Red represents positive correlation, blue represents negative correlation. **, *p* values < 0.01; *, *p* values < 0.05. (**A**) Differential accumulated metabolites (DAMs) and differentially expressed genes (DEGs) correlation analysis. Each row represents one DAM, and each column represents one DEG. (**B**) Enzymatic, non-enzymatic, and antioxidant indicator correlation analysis.

To elucidate the relationship between DAMs, DEGs, and antioxidant activity across various fermentation durations (D24, D30, D36), we standardized all data and conducted a correlation analysis. The results indicate that with the exception of genes *SPBC29A3.09c* and *YBT1*, the remaining DEGs exhibited significant or extremely significant positive or negative correlations with DAMs. This suggests that the fluctuations in the content of these metabolites are regulated, either directly or indirectly, by these genes (Figure 7A). Furthermore, these DAMs and DEGs demonstrated significant or extremely significant positive or negative correlations with four antioxidant indicators (FRAP, DPPH, •OH, O_2_^•−^). A noteworthy correlation was observed between these metabolites, enzymatic (SOD, POD, CAT, GSH-Px), and non-enzymatic (flavonoid, polysaccharide) antioxidants, which also showed significant or extremely significant positive or negative correlations (Figure S9A,B). Furthermore, both enzyme and non-enzyme indicators exhibited a highly significant positive correlation with the four antioxidant indicators (Figure 7B). This correlation suggests that an increase in the content of DEGs and DAMs promotes an increase in the levels of enzyme and non-enzyme substances, ultimately enhancing the antioxidant capacity of *O. sinensis*.

#### 3.4.2. Key Path Analysis

To elucidate the key metabolic pathways associated with the comparisons of D24 vs. D30, D24 vs. D36, and D36 vs. D30, the DAMs and DEGs from these three groups were mapped to the KEGG database. The analysis revealed that D24 vs. D30, D24 vs. D36, and D36 vs. D30 corresponded to 47, 70, and 72 metabolic pathways, respectively (Table S2). Considering gene *p* values and metabolite *p* values, the top 20 KEGG pathways with *p* < 0.05 were selected for further analysis. The findings indicate that the DAMs and DEGs in the D24 vs. D30 comparison were predominantly enriched in three metabolism pathways, fructose and mannose, glutathione, and tryptophan. In contrast, the DAMs and DEGs in the D24 vs. D36 comparison were primarily enriched in pathways such as the biosynthesis of amino acids, glyoxylate and dicarboxylate metabolism, and ABC transporters. The DAMs and DEGs of the D36 vs. D30 comparison were primary enriched in the ABC transporters, fructose and mannose metabolism, and pantothenate and CoA biosynthesis, among others. Screening revealed that seven pathways were common across the three comparison groups and exhibited significant differences (*p* < 0.05). These pathways include metabolic pathways, ABC transporters, glutathione metabolism, tryptophan metabolism, carbon metabolism, biosynthesis of secondary metabolites, and phenylalanine metabolism.

**Figure 8:**
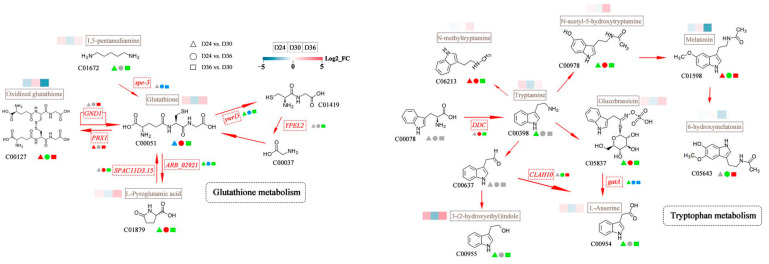
Overview of differential accumulated metabolites (DAMs) and differentially expressed genes (DEGs) mapping to key Kyoto Encyclopedia of Genes and Genomes (KEGG) pathways in pairwise comparisons of D24 vs. D30, D24 vs. D36, and D36 vs. D30. Red triangles indicate significant up-regulation between groups; green triangles indicate significant down-regulation; blue triangles indicate both up-regulation and down-regulation; gray triangles represent insignificant differences in detected DAMs or differentially expressed genes (DEGs). The other shapes have the same color explanation as before. Solid arrows represent facilitation, gray solid boxes represent DAMs, and red dotted boxes represent DEGs.

We selected two metabolism pathways, such as glutathione and tryptophan, that exhibited a high enrichment of DAMs and DEGs for further analysis. The aim was to elucidate the role of DAMs and DEGs in regulating the antioxidant activity of *O. sinensis*. The results indicate that in the glutathione metabolism pathway, the metabolites L-pyroglutamic acid, glutathione, and 1,5-pentanediamine were all down-regulated on D30. Notably, glutathione is regulated by an up-regulated transcription factor (*PRX1*), leading to a significant up-regulation of the oxidized form of glutathione on D30. In the tryptophan metabolism pathway, we observed that with the exception of the metabolite melatonin, which was significantly up-regulated on D30, most other metabolites were down-regulated at this time point (Figure 8).

### 3.5. Confirmation of DEGs Via qRT-PCR

**Figure 9:**
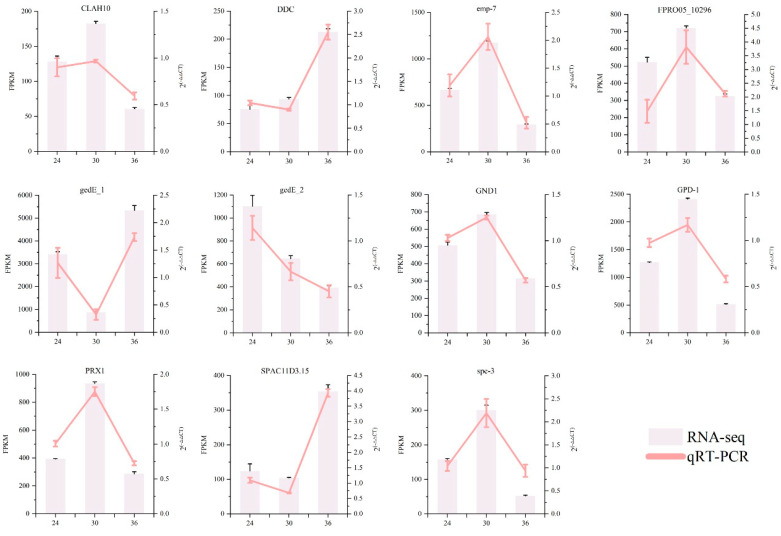
The qRT-PCR validation of 11 differentially expressed genes (DEGs).

To verify the stability and reliability of the transcriptome data, 11 DEGs associated with glutathione metabolism, tryptophan metabolism, carbon metabolism, and other pathways were selected for qRT-PCR verification. The results indicate that the qRT-PCR findings are consistent with the RNA-Seq results. Although the fold differences vary, the overall trend remains largely unchanged, suggesting that the transcriptome data are reliable (Figure 9).

## 4. Discussion

In recent years, *O. sinensis* has garnered significant attention, owing to its active ingredients and pharmacological effects, which are comparable to those of wild Chinese cordyceps. However, it is important to note that these pharmacological effects may vary depending on changes in growth conditions, environmental factors, and storage duration [45]. Research indicates that *O. sinensis* can produce a variety of metabolites, including polysaccharides, phenols, and flavonoids, during the fermentation process. Furthermore, the optimization of fermentation conditions, such as temperature, pH value, and fermentation time, also influences the accumulation of these metabolites [48,49,50,51]. This study utilized FRAP as the primary antioxidant index while also considering DPPH, •OH, and O_2_^•–^. The results indicate that the antioxidant capacity of *O. sinensis* initially increased and then decreased with prolonged fermentation time, with FRAP, DPPH, and •OH antioxidant activities reaching their maximum values on D30. Previous research has demonstrated that the composition of the *O. sinensis* fermentation medium was optimized to achieve a higher polysaccharide content [52]. Additionally, the melanin content reached its maximum on the 35th day of *O. sinensis* fermentation, exhibiting strong DPPH free radical scavenging ability and effective chelation of ferrous ions [53].

Reactive oxygen species (ROS) in the human body play a crucial role in cell signal transduction and the maintenance of cellular functions. However, when the concentration of ROS surpasses a certain threshold, it can lead to oxidative damage to cells, thereby contributing to the development of various diseases [11,12,13]. SOD, POD, CAT, and GSH-Px function as antioxidant enzymes that scavenge ROS produced within organisms, thereby preventing oxidative damage [54]. Flavonoids and polysaccharides, which can be sourced from plants, bacteria, and fungi, also serve as antioxidants. These compounds play a crucial role in reducing oxidative stress and preventing disease [23,24]. It has been reported that *O. sinensis* alleviates the oxidative stress induced by pulmonary fibrosis by enhancing the activities of SOD and GSH-Px in mice [55]. In addition to its immune effects [41], polysaccharides of *O. sinensis* can also up-regulate the expression levels of CAT, SOD, and methionine sulfoxide reductase (MTH), thereby extending the lifespan of Drosophila [56]. The FRAP antioxidant capacity of *Floccularia luteovirens* was positively correlated with flavonoid content [57]. Moreover, we observed that the concentrations of these substances consistently increased from D24, reached their maximum values on D30, and subsequently declined by D33. This pattern parallels the characterization of the antioxidant activity of *O. sinensis*. Similar phenomena were also reported in a previous study of *cordyceps militaris* [58]. Early studies have indicated that the subculture of fungi ultimately resulted in an increase in ROS concentrations [59]. Consequently, alterations in the antioxidant enzymes and antioxidant activities of *O. sinensis* may be associated with an imbalance in ROS levels.

Metabolomics is a powerful analytical technique that enables the comprehensive analysis and identification of metabolites in organisms. This method has been extensively utilized to compare the metabolite profiles of cordyceps fungi across different geographical locations, species, and cultural environments [60,61]. In this study, a total of 1784 metabolites were detected from samples collected on D24, D30, and D36. The four supercategories that were significantly enriched included lipids and lipid-like molecules, organic acids and derivatives, organoheterocyclic compounds, and benzenoids. This is consistent with the findings reported in references [62,63,64]. Previous studies have indicated that a reduction in the number of DAMs may be associated with the number of subcultures [65,66]. Through PCA and OPLS-DA, we identified 366 up-regulated DAMs on D24, 501 up-regulated DAMs on D30, and 312 up-regulated DAMs on D36. These findings suggest that the differences in metabolites of *O. sinensis* may be related to fermentation time. We identified 462 common DAMs across the comparisons of D24 vs. D30, D24 vs. D36, and D36 vs. D30. The first three categories of these metabolites, following their classification, were lipids, organoheterocyclic compounds, and organic acids and derivatives. These findings provide a reference for our search for biomarkers that contribute to the differences in the antioxidant activity of *O. sinensis* at varying fermentation times. And numerous secondary metabolites derived from these compounds have demonstrated potential value in medical, dietary, and other applications. For example, linoleic acid, which is present in higher concentrations on D24 and D36, may serve as an enhancer of adaptive T cell therapy in tumor treatment, thereby improving the anti-tumor efficacy of these cells [67]. Additionally, derivatives of the glycerophospholipid subclass have been shown to reduce the incidence of Alzheimer’s disease by mitigating cellular oxidative stress [68].

High-throughput transcriptome sequencing technology offers effective approaches to address the functional gene issues closely associated with the biosynthesis of primary and secondary metabolites. Early studies have utilized high-throughput metabolomics in conjunction with RNA sequencing (RNA-seq) technology to investigate the significant alterations that occur in various primary and secondary metabolites in plants and mushrooms. These studies have successfully identified the DEGs associated with metabolite biosynthesis [65,69,70]. In this study, we conducted a transcriptome analysis on samples from D24, D30, and D36, screening for DEGs based on gene expression levels. The results indicate that the duration of fermentation significantly influences gene expression levels. Illumina RNA-Seq technology was employed to perform GO enrichment and KEGG enrichment analyses of the DEGs. The results of the GO enrichment analysis indicate that terms such as structural molecule activity, protein-containing complex, and immune system process were significantly up-regulated on D30, encompassing several up-regulated genes (*mrt4*, *RPS2*, *spn4*, and *mcm3*). *Mrt4* is a nucleo-cytoplasmic shuttling factor [71], and its deletion impacts pre-rRNA processing, a characteristic that is commonly observed in various strains that are affected by 60S ribosomal subunit biogenesis [72,73]. Minichromosome maintenance proteins (MCMs) are essential subunits of the prereplication complex and may function as DNA helicases during the S phase of the cell cycle [74]. Research indicates that these genes are widely distributed across eukaryotes and archaea [75]. Specifically, *MCM3* acts as a licensing factor in eukaryotic cells and is crucial for the initiation of DNA replication. Furthermore, studies have demonstrated that low-level laser irradiation can enhance DNA replication and may stimulate osteoblast proliferation by increasing *MCM3* gene expression [76]. However, the abnormal expression and activation of MCMs often leads to the development of various malignant tumors and contributes to unstable and uncontrolled cell cycle progression within the genome [77]. Consequently, we observed that the terms significantly up-regulated on D30 were notably enriched and subsequently down-regulated on D36.

The KEGG enrichment analysis suggests that D24, D30, and D36 involve distinct metabolite pathways, potentially related to their DAMs. The pathways exhibiting significant differences on D30 were predominantly related to pentose and glucuronate interconversions, galactose metabolism, phenylalanine metabolism, pantothenate and CoA biosynthesis, and tyrosine metabolism. A classification of these pathways reveals that they can be primarily divided into categories of carbohydrates and amino acids. Polysaccharides, recognized as one of the four fundamental substances that constitute life, are present not only in animals and plants but also in edible and medicinal fungi [78]. They play a crucial role in numerous important biological processes, including intercellular communication, embryonic development, and cellular immunity [79,80,81]. Studies have demonstrated that *O. sinensis* polysaccharides can activate macrophage RAW264.7 via the MAPK and PI3K/Akt signaling pathways, thereby enhancing its immunomodulatory activity [82]. Additionally, they exhibit significant hydroxyl radical scavenging ability, reducing power, and Fe (2+)-chelating activity [83]. Amino acids are fundamental components of proteins and peptides, serving as precursors for the synthesis of various secondary metabolites [84]. Variations in the types and concentrations of amino acids significantly influence the quality of organisms [85]. Research indicates that deficiencies in amino acids can impede protein synthesis and hinder organismal development, resulting in symptoms such as memory loss, weakness, and swelling [86,87]. In plants, a deficiency of amino acids may lead to root rot and stunted growth, while in fungi, it can cause metabolic imbalances [88]. We found higher levels of amino acid metabolites contained on D24, D30, and D36. L-glutamic acid, a significant metabolite among amino acids, has been shown to effectively alleviate oxidative stress in rats [89]. DL-glutamic acid, the conjugate acid of L-glutamic acid, was found to be significantly up-regulated on D30, while it was down-regulated on D24 and D36.

The results of the correlation analysis between the metabolome and transcriptome indicate that the DAMs and DEGs were predominantly enriched in pathways related to glutathione metabolism, tryptophan metabolism, phenylalanine metabolism, carbon metabolism, and the biosynthesis of secondary metabolites. In the context of glutathione metabolism, we observed that one gene (*PRX1*) was significantly up-regulated on D30 and exhibited a notable positive correlation with both oxidized glutathione and reduced glutathione. *PRX1* exhibits CAT-like activity [90], which mitigates damage to plants under harsh environmental conditions, thereby serving a protective role [91]. Glutathione, an essential dietary supplement, possesses antioxidant, protective, and regulatory functions [92]. Elevated levels of oxidized glutathione have been shown to enhance skin characteristics, promote whitening, and delay the aging process [93,94]. The antioxidant activity of *O. sinensis* on D30 reached the maximum value that is associated with the interaction between *PRX1*, glutathione, and oxidized glutathione. Furthermore, we observed that the metabolite 5-oxoproline was significantly up-regulated at D36; however, its increased levels may contribute to oxidative stress [95]. The antioxidant activity of *O. sinensis* was decreased on D36, which may be associated with 5-oxoproline. Melatonin, recognized as an effective free radical scavenger, has its secretion influenced by the duration of darkness [96]. Furthermore, administration of high-dose melatonin (20 mg per subject) has been shown to significantly reduce serum lipid peroxidation products and levels of inflammation markers, thereby improving the survival rate of neonates with sepsis [97]. In tryptophan metabolism, we observed that the metabolite melatonin was significantly up-regulated on D30 but exhibited down-regulation on D36. Furthermore, the gene *DDC* in this metabolic pathway was significantly up-regulated on D36. Given that *DDC* increases ROS and oxidative stress kinase [98], we speculate that the down-regulation of melatonin on D36 may be influenced by the activity of the gene *DDC*. Glucose, as a high-carbohydrate compound, provides the energy required by the body and can be rapidly absorbed. Upon entering cells, glucose is converted into d-gluconic acid through the action of soluble NADP+-dependent glucose dehydrogenase [99,100]. D-gluconic acid is characterized as a non-corrosive, non-volatile, non-toxic, and mild organic acid. Its production and consumption are influenced by the specific strain, culture medium, and culture conditions [101,102]. Sainz et al. observed that the concentration of d-gluconic acid reached its maximum value on the 5th day of fermentation across three distinct media, and that this concentration varied with changes in pH levels [103]. In carbon metabolism, we observed that d-gluconic acid was significantly up-regulated on D30, followed by a down-regulation on D36. Additionally, glucose derivatives exhibit inhibitory effects on peroxynitrite-induced plasma lipid peroxidation [104]. Therefore, we hypothesize that the reduction in antioxidant activity of *O. sinensis* on D36 may be associated with the decreased levels of the glucose secondary metabolite d-gluconic acid.

## 5. Conclusions

This study employed FRAP as an indicator and comprehensively assessed DPPH, •OH, and O_2_^•−^ to evaluate the antioxidant activity of *O. sinensis*. The results indicate that the antioxidant activity of *O. sinensis* reached its maximum value on D30. The metabolic pathway map reveals that DAMs and DEGs influencing the antioxidant activity of *O. sinensis* were significantly up-regulated on D30, but down-regulated on D36. The correlation analysis suggests that an increase in the content of DEGs and DAMs promotes an increase in the levels of enzyme and non-enzyme substances, ultimately enhancing the antioxidant capacity of *O. sinensis*. In summary, these findings offer valuable insights into the impact of DAMs and DEGs on the antioxidant activity of *O. sinensis*, aiming to enhance the development and application of *O. sinensis*.

## Figures and Tables

**Table 1:** Gradient separation procedure.

Time (min)	Mobile Phase B
0–0.5	95%
0.5–7	65%
7–8	40%
8–9	40%
9–9.1	95%
9.1–12	95%

## Data Availability

The raw data were submitted to the National Center for Biotechnology Information Sequence Read Archive (NCBI SRA) (accession number: PRJNA1125860).

## Supplementary Materials

The following supporting information can be downloaded at https://www.mdpi.com/article/10.3390/jof11010051/s1, Figure S1: Total ion chromatograms (TIC). (A) TIC of QC (quality control) with untargeted metabolomics LC-MS/MS technology. (B) TIC of QC, D24, D30, and D36. Figure S2: Multivariate statistical analyses of metabolites detected on D24, D30, and D36. (A) OPLS-DA scores of D24, D30, and D36. (B) OPLS-DA permutation test of D24, D30, and D36. Figure S3: The DAMs analysis of the D24, D30, and D36. Superclass classification of DAMs in the pairwise comparison between D24 and D30, D24 and D36, and D30 and D36. Figure S4: Changes in the peak area of DAMs for different superclasses. The DAMs of (A) lipids and lipid-like molecules, (B) organic acids and derivatives, (C) organoheterocyclic compounds, (D) benzenoids, (E) organic oxygen compounds detected in O. sinensis at different fermentation times (D24, D30, and D36). The horizontal coordinate represents the DAMs, and the vertical coordinate is the peak area of the DAMs. Figure S5: Enrichment analysis of DMs between D24, D30, and D36 using the KEGG database. The top 20 pathways with the lowest p value were mapped, the vertical coordinate is the pathway, the horizontal coordinate is the enrichment factor (the number of differential metabolites in this pathway divided by all the quantities in this pathway), the size represents the quantity, and the redder the color, the higher the p value. (A) D24 vs. D30, (B) D24 vs. D36, (C) D36 vs. D30. Figure S6: GO annotation analysis of 3 comparison groups (A) D24 vs. D30, (B) D24 vs. D36, (C) D36 vs. D30. Figure S7: KEGG enrichment analysis of 3 comparison groups. (A) D24 vs. D30, (B) D24 vs. D36, (C) D36 vs. D30. Figure S8: The DAMs analysis of the three samples. Volcano plots of D24 vs. D30, D24 vs. D36, and D36 vs. D30. Figure S9. Correlation clustering heatmap. Red represents positive correlation, blue represents negative correlation. **, *p* values < 0.01; *, *p* values < 0.05. (A) Differential accumulated metabolites (DAMs), enzymatic and, non-enzymatic correlation analysis. (B) Differentially expressed genes (DEGs), enzymatic and, non-enzymatic correlation analysis. Table S1: Transcriptional sequencing data and quality assessment. Table S2: KEGG pathway in the three comparison groups under combined metabolomic and transcriptomic analysis. Table S3: The FPKM expression of differentially expressed genes (DEGs) in three samples.
