# Supplementary material for: Metabolomics and Transcriptomics Reveal the Effects of Different Fermentation Times on Antioxidant Activities of Ophiocordyceps sinensis
Source: J Fungi (Basel). 2025 Jan 9;11(1):51. doi: 10.3390/jof11010051 (PMC11766798; doi:10.3390/jof11010051)
Supplement: Supplementary file 1 [file jof-11-00051-s001.zip › Supplementary Figures.pdf]

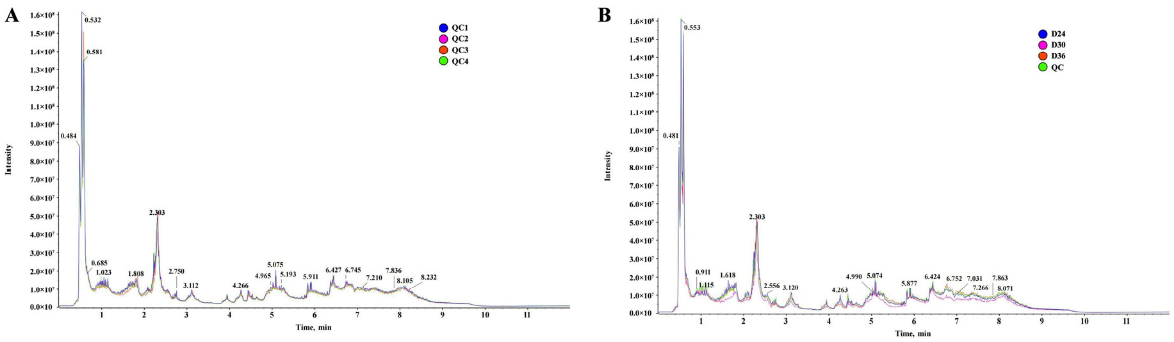

Figure S1. Total ion chromatograms (TIC). (**A**) TIC of QC (quality control) with untargeted metabolomics LC-MS/MS technology. (**B**) TIC of QC, D24, D30, and D36.

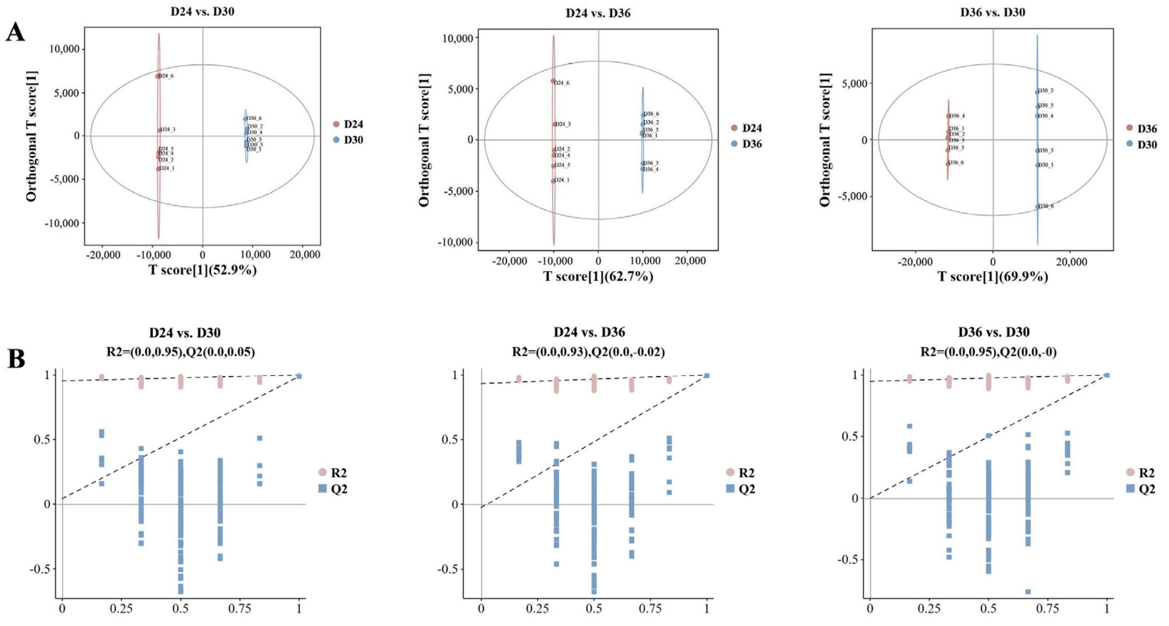

Figure S2. Multivariate statistical analyses of metabolites detected in D24, D30, and D36. (**A**) OPLS-DA scores of D24, D30, and D36. (**B**) OPLS-DA permutation test of D24, D30, and D36.

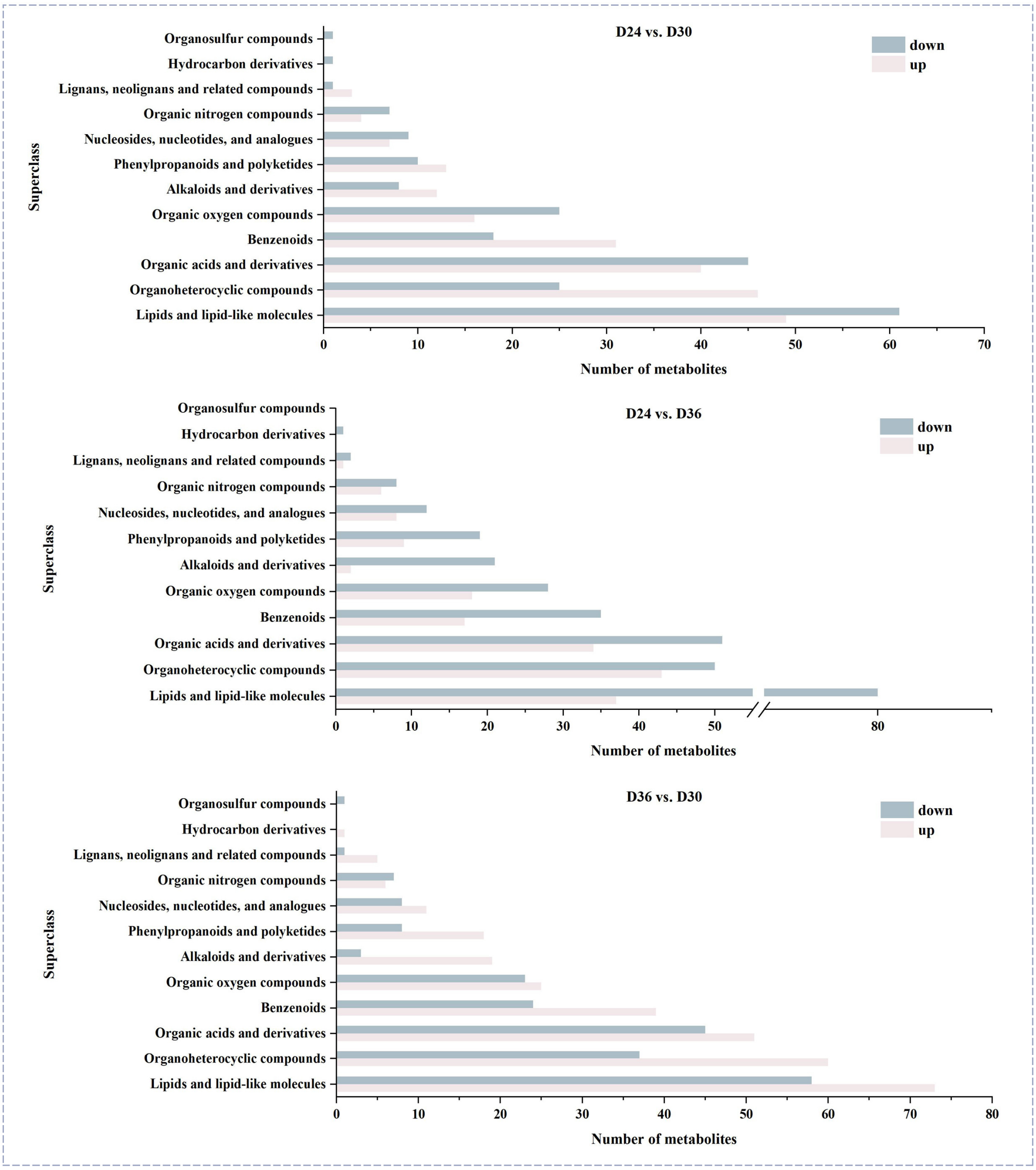

Figure S3. The DAMs analysis of the D24, D30, and D36.Superclass classification of DAMs in the pairwise comparison between D24 and D30, D24 and D36, and D30 and D36.

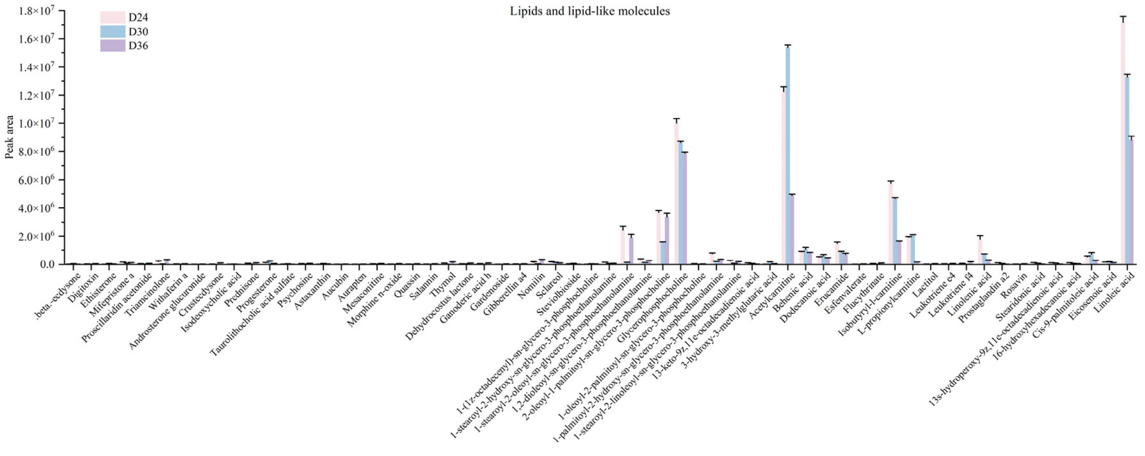
Lipids and lipid-like molecules
D24
D30
D36
Peak area
1.8×10^7
1.6×10^7
1.4×10^7
1.2×10^7
1.0×10^7
8.0×10^6
6.0×10^6
4.0×10^6
2.0×10^6
0.0
.beta.-ecdysone
Digitoxin
Ethisterone
Mifepristone a
Proscillaridin acetonide
Triamcinolone
Withaferin a
Androsterone glucuronide
Crustecdysone
Isodeoxycholic acid
Prednisone
Progesterone
Taurolithocholic acid sulfate
Psychosine
Astaxanthin
Aucubin
Aurapten
Mesaconitine
Morphine n-oxide
Quassin
Salannin
Thymol
Dehydrocostus lactone
Ganoderic acid h
Gardenoside
Gibberellin a4
Nomilin
Sclareol
Stevioside
Acetylcarnitine
Behenic acid
Dodecanoic acid
Erucamide
Esfenvalerate
Fluorthrinate
Isobutyryl-l-carnitine
L-propionylcarnitine
Lactitol
Leukotriene e4
Leukotriene f4
Linolenic acid
Prostaglandin a2
Rosavin
Stearidonic acid
16-hydroxyhexadecanoic acid
Cis-9-palmitoleic acid
Eicosenoic acid
Linoleic acid

(**A**)

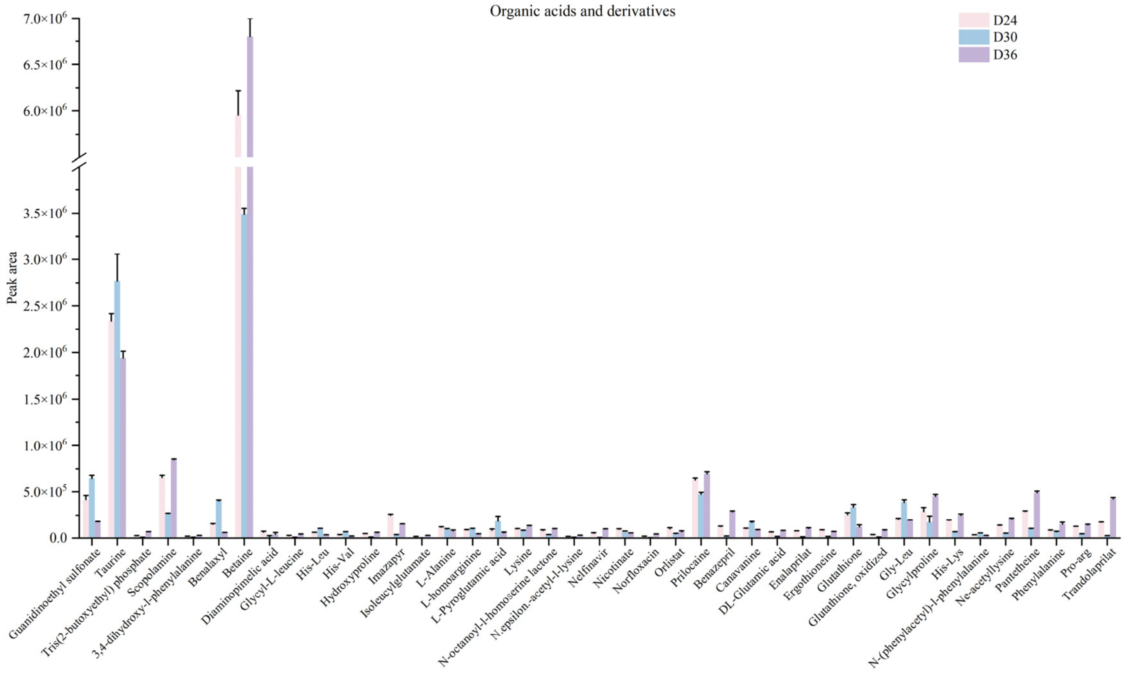
Organic acids and derivatives
D24
D30
D36
Peak area
7.0×10^6
6.5×10^6
6.0×10^6
3.5×10^6
3.0×10^6
2.5×10^6
2.0×10^6
1.5×10^6
1.0×10^6
5.0×10^5
0.0
Guanidinoethyl sulfonate
Taurine
Tris(2-butoxyethyl) phosphate
Scopolamine
3,4-dihydroxy-l-phenylalanine
Benalaxyl
Betaine
Diaminopimelic acid
Glycyl-L-leucine
His-Leu
His-Val
Hydroxyproline
Imazapyr
Isoleucylglutamate
L-Alanine
L-homoarginine
L-Pyroglutamic acid
Lysine
N-octanoyl-l-homoserine lactone
N.epsilon.-acetyl-l-lysine
Nelfinavir
Nicotinate
Norfloxacin
Orlistat
Prilocaine
Benazepril
Canavanine
DL-Glutamic acid
Enalaprilat
Ergothioneine
Glutathione
Glutathione, oxidized
Gly-Leu
Glycylproline
His-Lys
N-(phenylacetyl)-l-phenylalanine
Ne-acetyllysine
Pantetheine
Phenylalanine
Pro-arg
Trandolaprilat

(**B**)

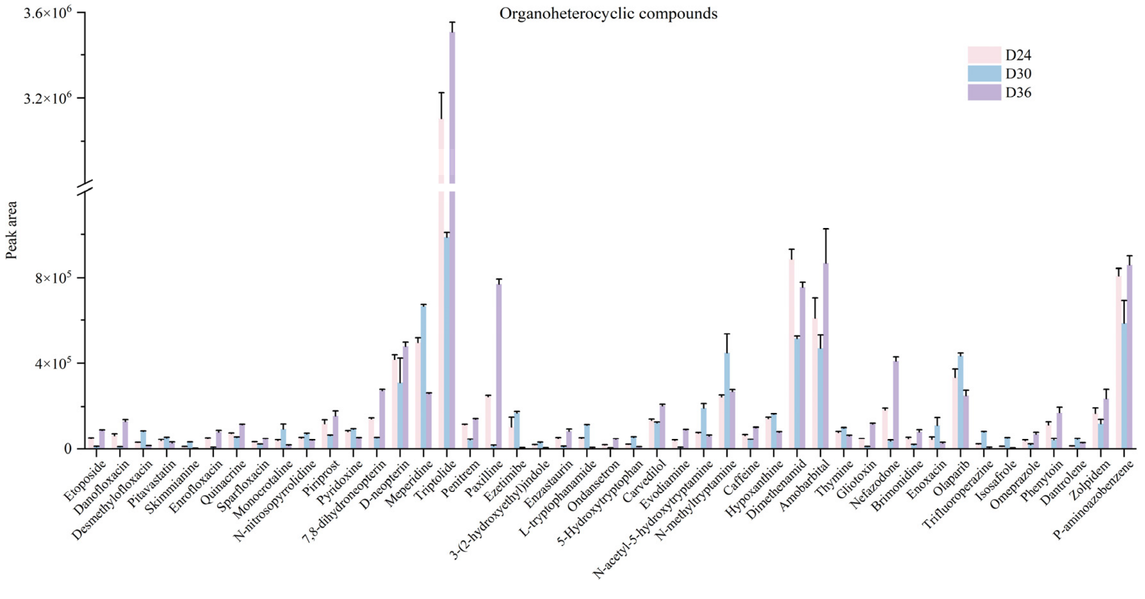
Organoheterocyclic compounds
D24
D30
D36
Peak area
3.6×10^6
3.2×10^6
8×10^5
4×10^5
0
Etoposide
Danofloxacin
Desmethylofloxacin
Pitavastatin
Skimmianine
Enrofloxacin
Quinacrine
Sparfloxacin
Monocrotaline
N-nitrosopyrrolidine
Piriprost
Pyridoxine
7,8-dihydroneopterin
D-neopterin
Meperidine
Triptolide
Penitrem
Paxilline
Ezetimibe
3-(2-hydroxyethyl)indole
Enzastaurin
L-tryptophanamide
Ondansetron
5-Hydroxytryptophan
Carvedilol
Evodiamine
N-acetyl-5-hydroxytryptamine
N-methyltryptamine
Caffeine
Hypoxanthine
Dimethenamid
Amobarbital
Thymine
Gliotoxin
Nefazodone
Brimonidine
Enoxacin
Olaparib
Trifluoperazine
Isosafrole
Omeprazole
Phenytoin
Dantrolene
Zolpidem
P-aminoazobenzene

(**C**)

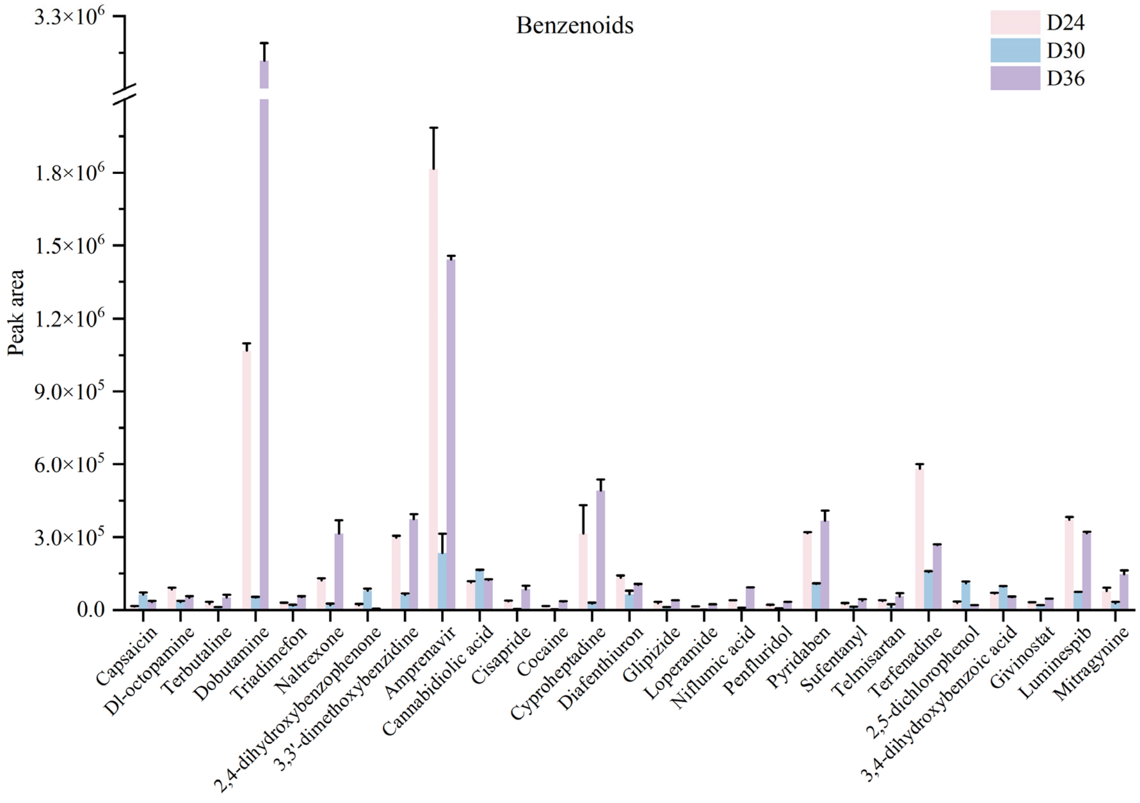

(**D**)

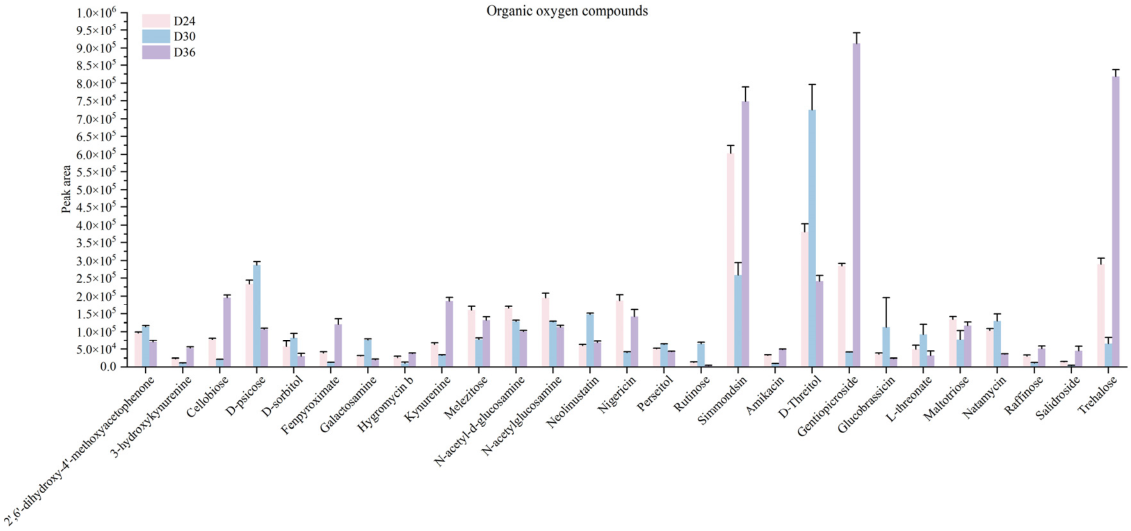

(**E**)

Figure S4. Changes in the peak area of DAMs for different superclasses. The DAMs of **(A)** lipids and lipid-like molecules, **(B)** organic acids and derivatives, **(C)** organoheterocyclic compounds, **(D)** Benzenoids, **(E)** Organic oxygen compounds detected in *O. sinensis* at different fermentation times (D24, D30, and D36). The horizontal coordinate represents the DAMs, and the vertical coordinate is the peak area of the DAMs.

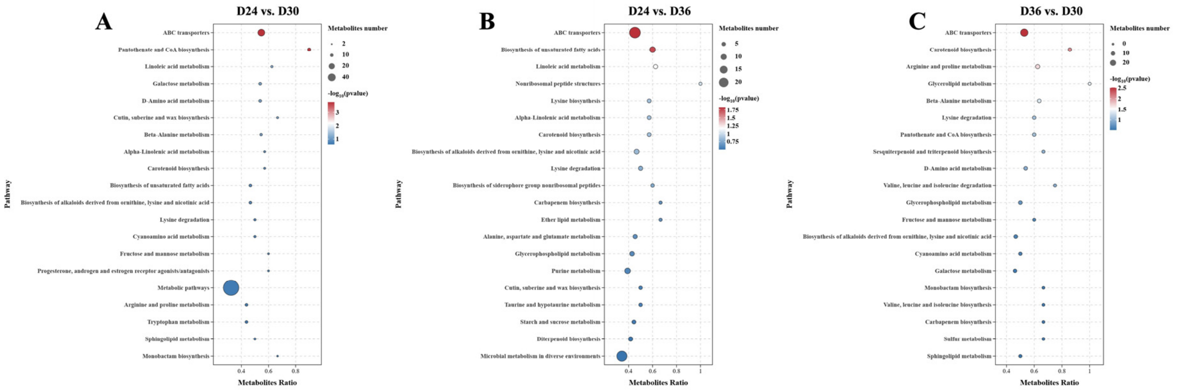

Figure S5. Enrichment analysis of DMs between D24, D30, and D36 using the KEGG database. The top 20 pathways with the lowest *P* value were mapped, the vertical coordinate is the pathway, the horizontal coordinate is the enrichment factor (the number of differential metabolites in this pathway divided by all the quantities in this pathway), the size represents the quantity, and the redder the color, the higher the *P* value. (**A**) D24 vs. D30, (**B**) D24 vs. D36, (**C**) D36 vs. D30.

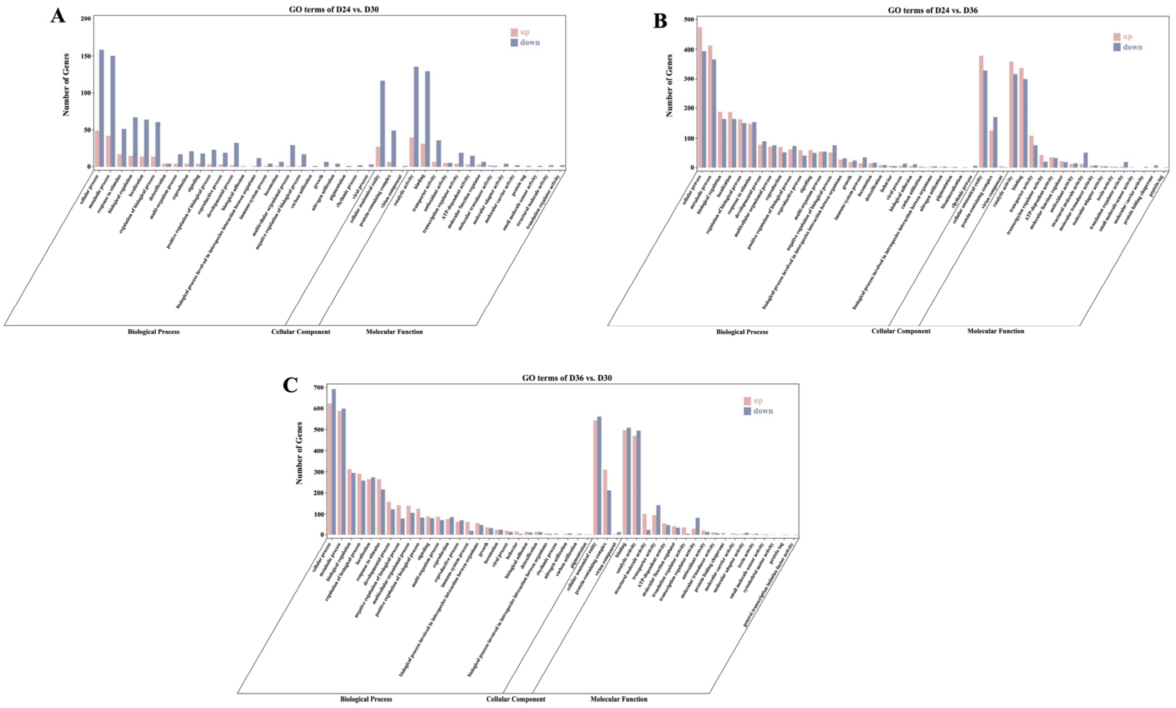

Figure S6. GO annotation analysis of 3 comparison groups (**A**) D24 vs. D30, (**B**) D24 vs. D36, (**C**) D36 vs. D30.

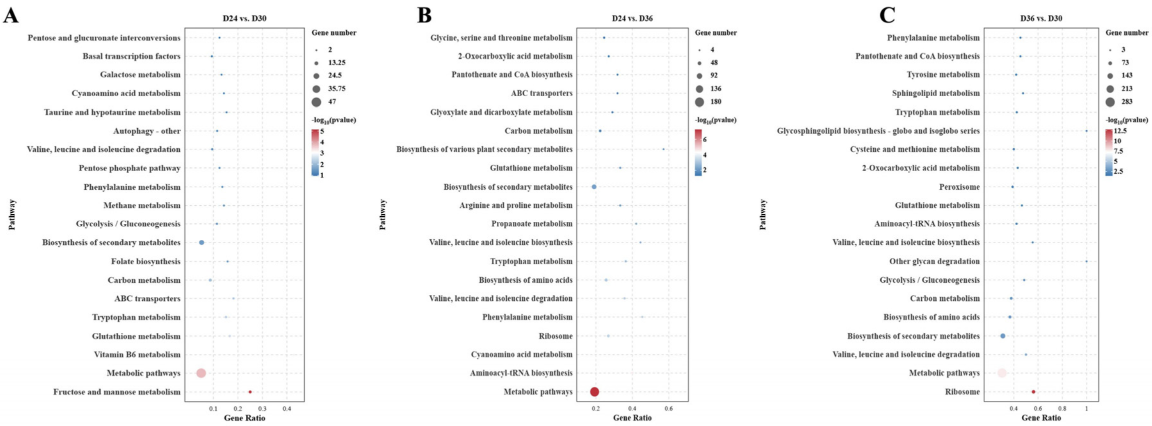

Figure S7. KEGG enrichment analysis of 3 comparison groups. (**A**) D24 vs. D30, (**B**) D24 vs. D36, (**C**) D36 vs. D30.

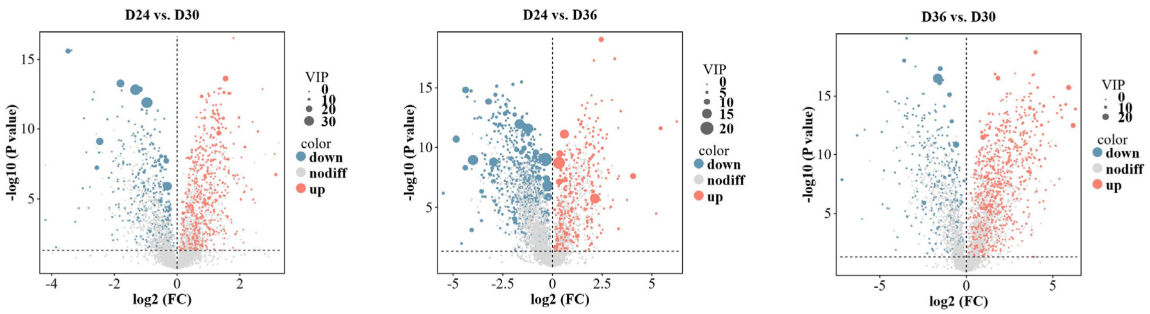

Figure S8. The DAMs analysis of the three samples. Volcano plots of D24 vs. D30, D24 vs. D36, and D36 vs. D30.

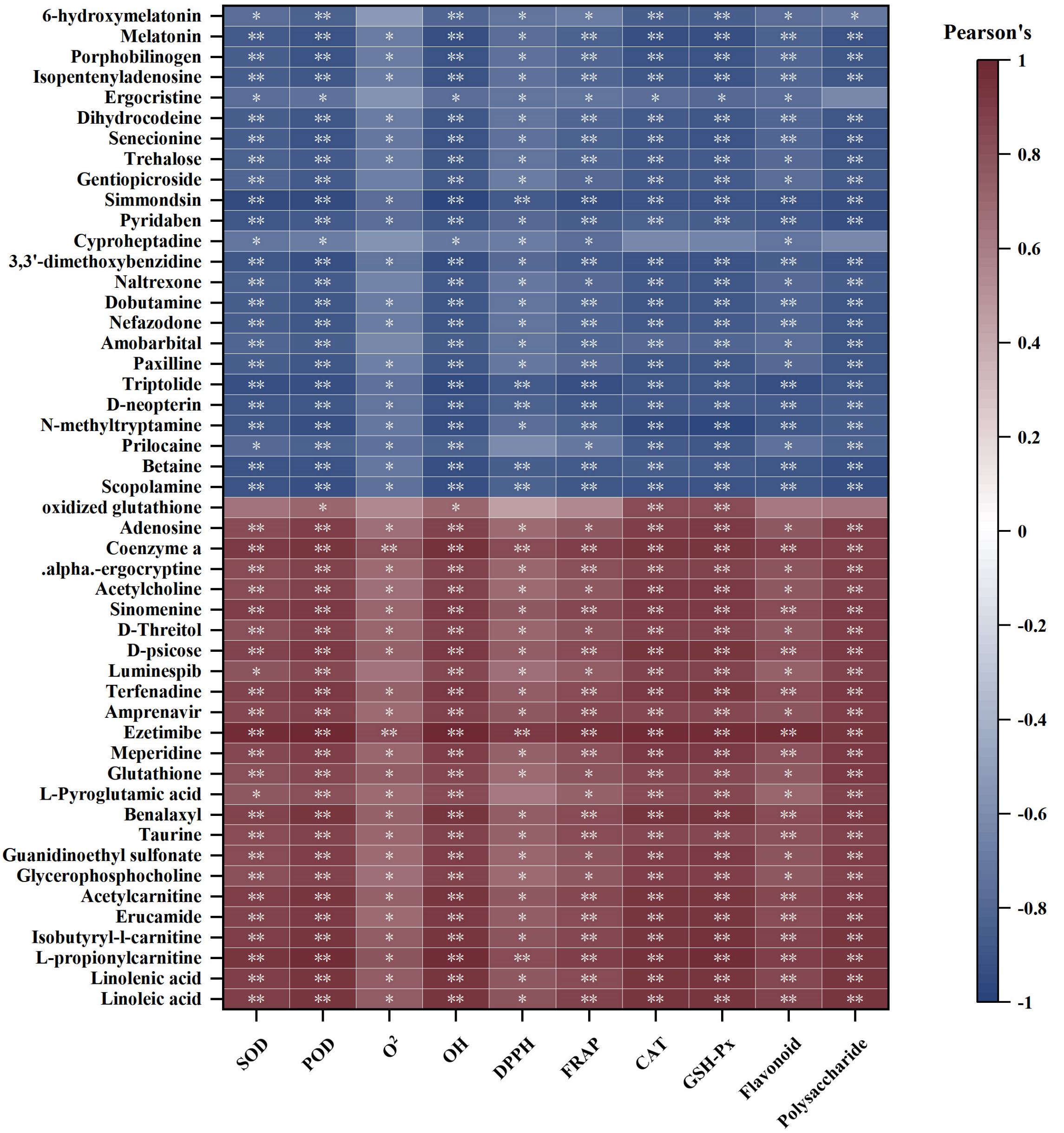

(**A**)

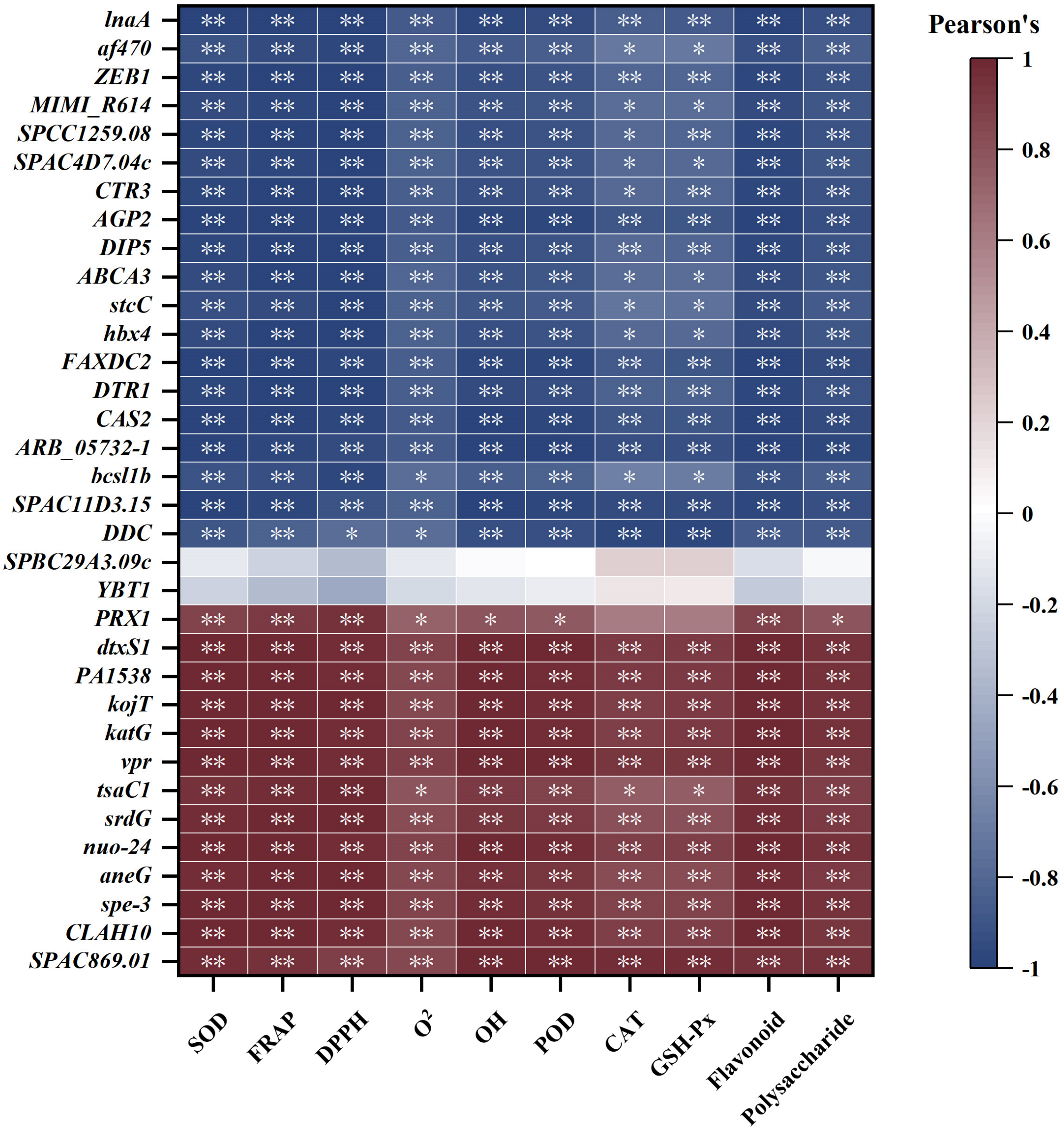

(B)

Figure S9. Correlation clustering heatmap. Red represents positive correlation, blue represents negative correlation. **, *p* values < 0.01; *, *p* values < 0.05. **(A)** Differential accumulated metabolites (DAMs), enzymatic and, non-enzymatic correlation analysis. **(B)** Differentially expressed genes (DEGs), enzymatic and, non-enzymatic correlation analysis.
